# STAT3/LKB1 controls metastatic prostate cancer by regulating mTORC1/CREB pathway

**DOI:** 10.1186/s12943-023-01825-8

**Published:** 2023-08-12

**Authors:** Jan Pencik, Cecile Philippe, Michaela Schlederer, Emine Atas, Matteo Pecoraro, Sandra Grund-Gröschke, Wen (Jess) Li, Amanda Tracz, Isabel Heidegger, Sabine Lagger, Karolína Trachtová, Monika Oberhuber, Ellen Heitzer, Osman Aksoy, Heidi A. Neubauer, Bettina Wingelhofer, Anna Orlova, Nadine Witzeneder, Thomas Dillinger, Elisa Redl, Georg Greiner, David D’Andrea, Johnny R. Östman, Simone Tangermann, Ivana Hermanova, Georg Schäfer, Felix Sternberg, Elena E. Pohl, Christina Sternberg, Adam Varady, Jaqueline Horvath, Dagmar Stoiber, Tim I. Malcolm, Suzanne D. Turner, Eileen E. Parkes, Brigitte Hantusch, Gerda Egger, Stefan Rose-John, Valeria Poli, Suneil Jain, Chris W. D. Armstrong, Gregor Hoermann, Vincent Goffin, Fritz Aberger, Richard Moriggl, Arkaitz Carracedo, Cathal McKinney, Richard D. Kennedy, Helmut Klocker, Michael R. Speicher, Dean G. Tang, Ali A. Moazzami, David M. Heery, Marcus Hacker, Lukas Kenner

**Affiliations:** 1 Department of Pathology, Medical University of Vienna, 1090 Vienna, Austria; 2 Center for Biomarker Research in Medicine, 8010 Graz, Austria; 3 Molecular and Cell Biology Laboratory, The Salk Institute for Biological Studies, La Jolla, CA 92037 USA; 4 Division of Nuclear Medicine, Department of Biomedical Imaging and Image-Guided Therapy, Medical University of Vienna, 1090 Vienna, Austria; 5 Institute for Research in Biomedicine, Università Della Svizzera Italiana, 6500 Bellinzona, Switzerland; 6 Department of Biosciences and Medical Biology, Cancer Cluster Salzburg, Paris-Lodron University of Salzburg, 5020 Salzburg, Austria; 7 Department of Pharmacology & Therapeutics, Roswell Park Comprehensive Cancer Center, Buffalo, NY 14263 USA; 8 Experimental Therapeutics Graduate Program, Roswell Park Comprehensive Cancer Center, Buffalo, NY 14203 USA; 9 Department of Urology, Medical University Innsbruck, 6020 Innsbruck, Austria; 10 Unit for Pathology of Laboratory Animals, University of Veterinary Medicine Vienna, 1210 Vienna, Austria; 11 Central European Institute of Technology, Masaryk University, 60177 Brno, Czech Republic; 12 Christian Doppler Laboratory for Applied Metabolomics (CDL-AM), Medical University of Vienna, 1090 Vienna, Austria; 13 Institute of Human Genetics, Medical University of Graz, 8010 Graz, Austria; 14 Department for Basic and Translational Oncology and Hematology, Division Molecular Oncology and Hematology, Karl Landsteiner University of Health Sciences, 3500 Krems, Austria; 15 Institute of Animal Breeding and Genetics, University of Veterinary Medicine Vienna, 1210 Vienna, Austria; 16 Department of Laboratory Medicine, Medical University of Vienna, 1090 Vienna, Austria; 17 Department of Urology, Medical University of Vienna, 1090 Vienna, Austria; 18 Department of Molecular Sciences, Swedish University of Agricultural Sciences, 75007 Uppsala, Sweden; 19 Center for Cooperative Research in Biosciences, Basque Research and Technology Alliance (BRTA), 20850 Derio, Spain; 20 Department of Pathology, Medical University Innsbruck, 6020 Innsbruck, Austria; 21 Institute of Physiology, Pathophysiology and Biophysics, University of Veterinary Medicine, 1210 Vienna, Austria; 22 Biochemical Institute, University of Kiel, 24098 Kiel, Germany; 23 Institute of Pharmacology, Center for Physiology and Pharmacology, Medical University of Vienna, 1090 Vienna, Austria; 24 Division Pharmacology, Department of Pharmacology, Physiology and Microbiology, Karl Landsteiner University of Health Sciences, 3500 Krems, Austria; 25 Department of Pathology, University of Cambridge, Cambridge, CB20QQ UK; 26 Institute of Medical Genetics and Genomics, Faculty of Medicine, Masaryk University, Kamenice 5, 62500 Brno, Czech Republic; 27 Department of Oncology, University of Oxford, Oxford, OX37DQ UK; 28 Ludwig Boltzmann Institute Applied Diagnostics, 1090 Vienna, Austria; 29 Comprehensive Cancer Center, Medical University of Vienna, 1090 Vienna, Austria; 30 Department of Molecular Biotechnology and Health Sciences, Molecular Biotechnology Center, University of Turin, 10126 Turin, Italy; 31 Patrick G Johnston Centre for Cancer Research, Queen’s University Belfast, Belfast, BT71NN UK; 32 MLL Munich Leukemia Laboratory, 81377 Munich, Germany; 33 Université Paris Cité, INSERM UMR-S1151, CNRS UMR-S8253, Institut Necker Enfants Malades, 75015 Paris, France; 34 Almac Diagnostics, Craigavon, BT63 5QD UK; 35 School of Pharmacy, University of Nottingham, Nottingham, NG7 2RD UK

**Keywords:** STAT3, mTORC1, AR, Prostate Cancer, LKB1, AMPK, CREB, Metformin

## Abstract

Prostate cancer (PCa) is a common and fatal type of cancer in men. Metastatic PCa (mPCa) is a major factor contributing to its lethality, although the mechanisms remain poorly understood. PTEN is one of the most frequently deleted genes in mPCa. Here we show a frequent genomic co-deletion of PTEN and STAT3 in liquid biopsies of patients with mPCa. Loss of *Stat3* in a *Pten*-null mouse prostate model leads to a reduction of LKB1/pAMPK with simultaneous activation of mTOR/CREB, resulting in metastatic disease. However, constitutive activation of Stat3 led to high LKB1/pAMPK levels and suppressed mTORC1/CREB pathway, preventing mPCa development. Metformin, one of the most widely prescribed therapeutics against type 2 diabetes, inhibits mTORC1 in liver and requires LKB1 to mediate glucose homeostasis. We find that metformin treatment of STAT3/AR-expressing PCa xenografts resulted in significantly reduced tumor growth accompanied by diminished mTORC1/CREB, AR and PSA levels. PCa xenografts with deletion of STAT3/AR nearly completely abrogated mTORC1/CREB inhibition mediated by metformin. Moreover, metformin treatment of PCa patients with high Gleason grade and type 2 diabetes resulted in undetectable mTORC1 levels and upregulated STAT3 expression. Furthermore, PCa patients with high CREB expression have worse clinical outcomes and a significantly increased risk of PCa relapse and metastatic recurrence. In summary, we have shown that STAT3 controls mPCa via LKB1/pAMPK/mTORC1/CREB signaling, which we have identified as a promising novel downstream target for the treatment of lethal mPCa.

**Supplementary Information:**

The online version contains supplementary material available at 10.1186/s12943-023-01825-8.

## Introduction

Prostate cancer (PCa) is one of the leading causes of cancer-related death in men worldwide [1]. Loss of the androgen receptor (AR) and PTEN tumor suppressor gene are among the most common genetic changes seen in PCa [2]. The AR and PTEN genes are key regulators of prostate cell growth and development, and their loss can contribute to the development of PCa by promoting uncontrolled cell proliferation. Signal transducer and activator of transcription 3 (STAT3) is a complex transcriptional and metabolic regulator responsible for essential biological functions such as cell differentiation, proliferation, immune response, and metabolism [3]. STAT3 signaling has both, tumor-suppressive and oncogenic roles in specific tissue contexts and is implicated in the regulation of malignant transformation and metastatic dissemination [4]. For example, loss of STAT3 expression was found to synergize with driver mutations to promote brain [5] or melanoma metastasis [6], whereas in lung cancer and PCa mouse models, disruption of STAT3 suppressed lung or prostate cancers compared to the wild-type cohort [7–9]. STAT3 transcriptional activity can trigger a metabolic switch to aerobic glycolysis to regulate mitochondrial activity, a prominent metabolic feature of tumor cells [10–12].

The serine/threonine kinase mTOR is a master regulator of cell growth and metabolism and is negatively regulated by LKB1 (also known as serine/threonine kinase 11 or STK11) [13]. Activation of mTOR leads to phosphorylation of eukaryotic initiation factor 4E-binding protein-1 (4E-BP1) and promotes protein synthesis [14]. mTOR promotes the phosphorylation of many substrates directly or indirectly by activating downstream kinases, including ribosomal S6 kinase (S6K), and the subsequent phosphorylation of S6 ribosomal protein to stimulate protein translation and cell proliferation. mTOR signaling is also frequently activated in cancer and is associated with various diseases, including obesity, metabolic syndrome, and type 2 diabetes mellitus (t2DM) [15]. A wide range of mTORC1 inhibitors display antitumor activity in glioblastoma [16] and PCa patients [17]. However, PI3K/mTOR inhibition activates AR signaling, which has been proven to be negative prognostic factor in human xenografts and transgenic mouse models of PCa [2]. Clinical trials with mTORC1 inhibitors revealed that blocking mTORC1 is associated with non-negligible side effects and toxicity and therefore didn’t meet clinical trial expectations [18].

Several reasons might explain the limited impact of mTOR blockade, including heterogeneous intratumoral mTORC1 activity, resistance mutations of mTOR or upstream PI3K-AKT driver mutations, and activation of alternative proliferative signaling pathways [19]. The mTOR pathway regulates CREB by modulating the activity of S6K1 (ribosomal protein S6 kinase 1) [20], an enzyme that phosphorylates ribosomal protein S6 and stimulates CREB activity. Thus, the mTOR pathway indirectly influences CREB activity and gene expression.

Metformin, a first-line treatment for patients with t2DM, activates AMP-activated protein kinase (AMPK), which results in inhibition of mTORC1 [21]. Metformin inhibits glucose and glutamine utilization in the mitochondria and has been associated with decreased cancer risk in epidemiological studies in t2DM patients [22] and in a variety of diabetic animal models [23]. Large-scale observational studies have found associations between metformin use and improved survival from deadly cancers, such as colon, liver, and lung cancers [24]. Several studies have shown that metformin may inhibit the growth of PCa cells in vitro and in vivo and reduce the risk of PCa. However, the evidence for the use of metformin as a treatment for PCa remains controversial. Rothermundt et al*.* [25] found stabilization and prolongation of prostate-specific antigen (PSA) doubling time in 23 patients (52.3%) as well as a reduction in metabolic parameters after starting metformin treatment. To date, however, the beneficial effect of metformin in reducing PCa incidence and improving overall survival is debated, particularly regarding the mode of action of metformin in clinical dosing of tumors. The role of STAT3 activation in PCa initiation and progression is dependent on the mutational context. We have shown that STAT3 acts as a tumor suppressor upon PTEN loss, thus overcoming senescence [9]. Furthermore, numerous studies have shown that STAT3 activation regulates several important metabolic functions. STAT3 has been described as a master metabolic regulator that sustains the glycolytic [11] and oxidative phosphorylation [26] activities of cells. The tumor suppressor LKB1, a key upstream regulator of the metabolic sensor AMPK [27], also activates the tumor suppressor PTEN [28].

Recently, Hermanova et al. [29] found that the co-deletion of *Pten* and *Lkb1* (Stk11) leads to more aggressive PCa and the formation of lung metastases. Moreover, loss of LKB1 in T cells leads to hyperactivation of the JAK/STAT pathway [30], but there is little evidence for a direct role of STAT3 in t2DM PCa downstream of metformin.

In this study, we illuminate the functional relationships between STAT3 and PCa growth using *Pten*-null mouse prostate model. We found that STAT3 strongly bound the LKB1/STK11 promoter region, therefore controlling LKB1/AMPK-induced genes expression in PCa. Loss of STAT3 function leads to downregulation of LKB1/AMPK1, and constitutive activation of STAT3 leads to activation of LKB1/AMPK in an established PCa mouse model based on *Pten*-null in prostate epithelial cells. Consistent with mTOR as a key effector in the biology of LKB1, proteomic analyses of mouse PCa models confirmed that mTORC1/CREB activation is STAT3-dependent. Metformin therapy of PCa patients with poor prognosis and concurrent t2DM leads to a decrease in mTORC1/CREB expression. Metformin treatment of androgen-dependent PCa cells xenografted into mice significantly reduced tumor growth, AR and PSA expression, and mTORC1/CREB signaling. We also show that high CREB expression levels are strongly associated with the risk of biochemical and metastatic relapse in PCa patients. Finally, we associate STAT3 and CREB expression status with AR signaling and ADT resistance in PCa patients, supporting the hypothesis that these are critical regulators and therapeutic targets in metastatic PCa. Overall, we show that the STAT3 targets LKB1 and regulates metastatic PCa growth via mTORC1/CREB, which we reveal as a promising downstream treatment target for lethal mPCa.

## Results

### Loss of STAT3 accelerates metastatic progression and impairs LKB1/AMPK signaling

**Fig. 1:**
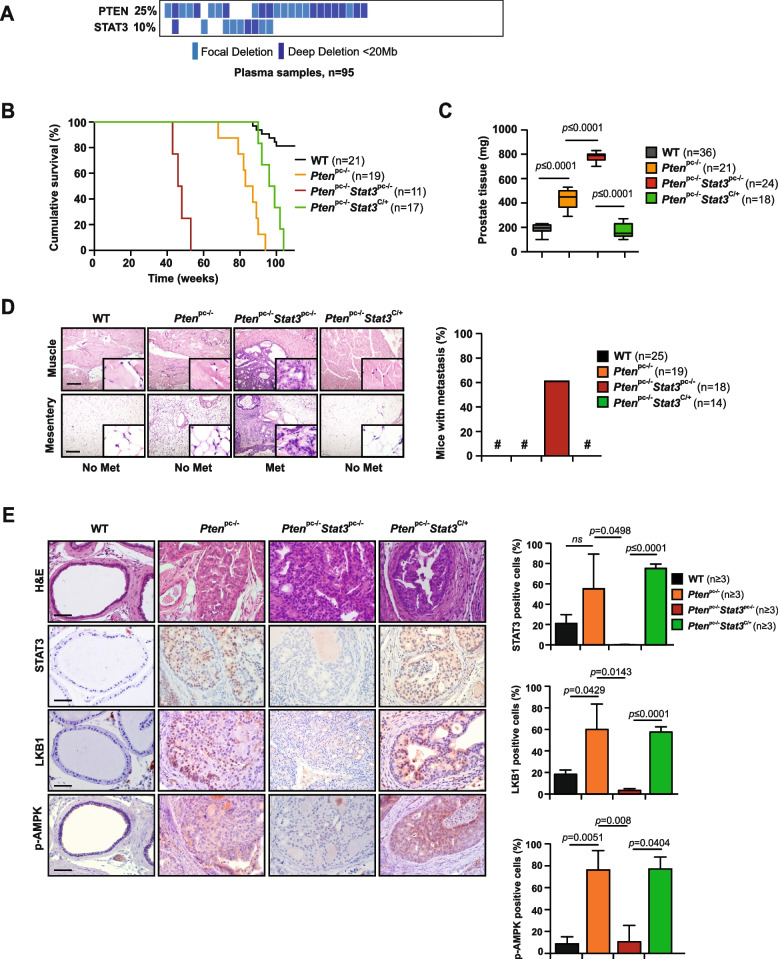
Loss of STAT3 accelerates metastatic progression and exhibits decreased LKB1/AMPK signaling. **A** High occurrence of *PTEN* and *STAT3* deletions in plasma samples of PCa patients with aggressive PCa (*n* = 95). **B** Kaplan–Meier cumulative survival analysis revealed a significant (*p* = 0.0026; log-rank test) increase in lifespan of *Pten*^*pc−/−*^*Stat3*^*C/*+^ compared to *Pten*^*pc−/−*^*Stat3*^*pc−/−*^ mice; WT and *Pten*^*pc−/−*^ mice served as controls (*n* = 68). **C** Prostate weights of 19-week-old WT, *Pten*^*pc−/−*^, *Pten*^*pc−/−*^*Stat3*^*pc−/−*^ and *Pten*^*pc−/−*^*Stat3*^*C/*+^ mice (*n* = 99). Mean values are shown; Data were analyzed by one-way analysis of variance with Tukey’s multiple comparison test; error bars: s.d. **D** IHC of muscle and mesentery, 52 weeks of age WT, *Pten*^*pc−/−*^, *Pten*^*pc−/−*^*Stat3*^*C/*+^ and *Pten*^*pc−/−*^*Stat3*^*pc−/−*^ mice. MET- metastasis, Scale bars, 100 μm; insets: × 600 magnification. Percentage of mice with distant PCa metastases (*n* = 76). **E** Haematoxilin/eosin (H&E) stains show only high-grade PIN in *Pten*^*pc−/−*^*Stat3*^*C/*+^ mice compared with *Pten*^*pc−/−*^ and *Pten*^*pc−/−*^*Stat3*^*pc−/−*^ mice. Scale bars, 100 μm. IHC analysis of Stat3, Lkb1 and p-Ampk in prostates from 19-week-old WT, *Pten*^*pc−/−*^, *Pten*^*pc−/−*^ *Stat3*^*pc−/−*^ and *Pten*^*pc−/−*^*Stat3*^*C/*+^ mice. Scale bars, 100 μm. Bar graphs indicate percentage of cells positive for Stat3, Lkb1 and p-Ampk in prostates of 19-week-old WT, *Pten*^*pc−/−*^, *Pten*^*pc−/−*^*Stat3*^*pc−/−*^ and *Pten*^*pc−/−*^*Stat3*.^*C/*+^. Protein levels quantification was done with HistoQuest software (n ≥ 3)

Mutation analysis revealed co-deletions of STAT3 and PTEN in DNA of patients with primary PCa and cfDNA plasma samples of patients with metastatic PCa (*n* = 95) [31] (Fig. 1A, Supplementary Fig. 1A). The selective pressure on STAT3 loss during metastatic progression suggests that this step may be required to facilitate tumor dissemination. To test this hypothesis and identify pathways that trigger the metastatic program of PCa cells, we utilized a metastatic PCa mouse model with *Stat3* and *Pten* deletions (*Pten*^*pc−/−*^ *Stat3*^*pc−/−*^), that develops aggressive disseminated PCa within 6 months [9]. To study the role of STAT3 in metastatic reprogramming, we utilized a constitutively active knock-in *Stat3C* allele replacing the endogenous WT allele [32]. The *Stat3*^*C/*+^ mice were crossed with the PCa mouse model of PTEN loss (*Pten*^*pc−/−*^*)* [9] to obtain *Pten*^*pc−/−*^ *Stat3*^*C/*+^ mice. Constitutive activation of *Stat3* in *Pten*^*pc−/−*^ mice significantly prolonged survival and decreased prostate tissue weight (Fig. 1B, C), in sharp contrast to *Pten*^*pc−/−*^ *Stat3*^*pc−/−*^ mice that died rapidly from metastatic disease. We could not find any evidence of tumor dissemination or metastasis in *Pten*^*pc−/−*^ *Stat3*^*C/*+^ mice (Fig. 1D) up to > 52 weeks of age, which suggests that activated *Stat3* plays a major role in preventing metastatic dissemination. Furthermore, *Pten*^*pc−/−*^ *Stat3*^*C/*+^ mice showed no visible signs of tumor growth (Supplementary Fig. 1B) and no histological sign of invasion but only minimal incidence of high-grade prostate intraepithelial neoplasia (PIN) (Fig. 1E), thereby impairing tumor progression and formation of metastasis. Furthermore, we discovered that *Pten*^*pc−/−*^*Stat3*^*pc−/−*^ tumor cells have high-grade mitochondrial structural damage that is not evident in *Pten*^*pc−/−*^*Stat3*^*C/*+^ prostate epithelial cells (Supplementary Fig. 1C). Similar inclusions have been described in defects in the assembly of the ATP synthase enzyme complex at the inner mitochondrial membrane, where inclusion bodies and loss of mitochondrial cristae occur [33].

Since loss of PTEN and LKB1 [29] is reminiscent of the phenotype observed for deletions of *Pten* and *Stat3* in PCa mouse models, we were prompted to investigate this further. The tumor suppressor function of LKB1 is attributed to activation of the energy sensor AMP-activated protein kinase (AMPK) in response to energy stress. The LKB1-AMPK axis shifts cellular metabolism from active anabolic to catabolic pathways, resulting in metabolic reprogramming [27, 34]. Importantly, we identified that *Pten*^*pc−/−*^ *Stat3*^*pc−/−*^ tumors displayed loss of STAT3, LKB1 and p-AMPK protein expression compared to *Pten*^*pc−/−*^ tumors (Fig. 1E). In contrast, in *Pten*^*pc−/−*^*Stat3*^*C/*+^ mice, STAT3, LKB1 and p-AMPK overall protein levels as well as the number of positive stained cells were markedly increased in comparison to *Pten*^*pc−/−*^ *Stat3*^*pc−/−*^ tumors (Fig. 1E) suggesting that STAT3 is a regulator of LKB1/AMPK signaling.

### STAT3 and LKB1 cooperate to suppress mTORC1

**Fig. 2:**
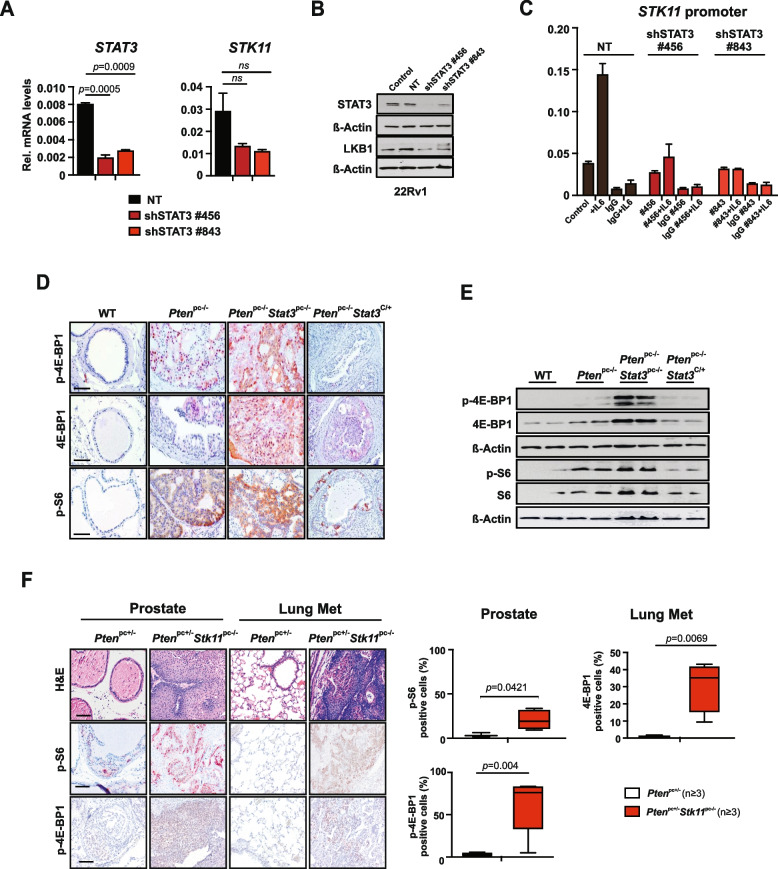
STAT3 and LKB1 cooperate to suppress mTORC1. **A** Western blot analysis of STAT3 and LKB1 in 22Rv1 cells transfected with non-targeting (NT) or shRNAs specific for STAT3 and/or LKB1. **B** qRT–PCR analysis of *STAT3* and *STK11* in 22Rv1 cells transfected with NT or shRNA specific for STAT3 (*n* = 3 each). **C** ChIP analysis of STAT3 binding to *LKB1 (STK11)* promoter. 22Rv1 cells harboring NT or two different shSTAT3 constructs were stimulated with IL-6 and immunoprecipated with STAT3 antibody or IgG as a negative control. Bars represent mean ± s.d. from 2 technical replicates. Precipitated DNA is presented as % of input. **D** IHC analysis of p-4E-BP1, 4E-BP1, p-S6 in prostates from 19-week-old WT, *Pten*^*pc−/−*^, *Pten*^*pc−/−*^ *Stat3*^*pc−/−*^ and *Pten*^*pc−/−*^*Stat3*^*C/*+^ mice. Scale bars, 100 μm. **E** Western blots analysis of p-4E-BP1, 4E-BP1, p-S6 and S6 expression in prostates from 19-week-old WT, *Pten*^*pc−/−*^, *Pten*^*pc−/−*^ *Stat3*^*pc−/−*^ and *Pten*^*pc−/−*^*Stat3*^*C/*+^ mice. β-actin serves as a loading control. **F** H&E and IHC analyses of Stat3, p-S6 and p-4E-BP1 in prostates and lung metastases from *Pten*^*pc*+*/−*^ and *Pten*^*pc*+*/−*^ *Stk11*^*pc−/−*^ mice, scale bars, 100 μm. Quantification of cells positive for STAT3 and p-4E-BP1 in 19-week-old *Pten*^*pc*+*/−*^ and *Pten*^*pc*+*/−*^ *Stk11*^*pc−/−*^ prostate tissue or lung metastases using HistoQuest software (*n* = 3). Data were analyzed by Student’s *t*-test and are shown as mean ± s.d

To further corroborate the functional role of STAT3 in regulation of LKB1/AMPK/mTORC1, we knocked down STAT3 expression in human PCa 22Rv1 cells. Notably, knockdown of *STAT3* caused decreased *LKB1 (STK11)* mRNA and protein expression (Fig. 2A, B). In addition, we analyzed passage-matched *Stat3*^*−/−*^ MEFs. Loss of *Stat3* resulted in reduced *LKB1 (STK11)* mRNA levels protein expression, consistent with mTORC1 upregulation (Supplementary Fig. 2A, B). indicating a possible STAT3 mediated transcriptional regulation of LKB1. Indeed, we confirmed a putative STAT3/consensus gamma interferon activation site (GAS) present in the *LKB1* (*STK11*) promoter using the Transcription Factor Affinity Prediction software (TRAP) as also validated by Linher-Melville et al*.* [35]. We performed a ChIP assay using an antibody against STAT3 in control versus STAT3 knockdown 22RV1 cells stimulated with IL-6. These results confirmed that endogenous STAT3 binds to the predicted regions on the *LKB1 (STK11)* promoter and that *LKB1 (STK11)* is a direct target gene of STAT3 (Fig. 2C).

One of the major growth regulatory pathways controlled by LKB1 (STK11) is the mTOR pathway. LKB1 activates AMPK, which then rapidly inhibits a central integrator of cell metabolism and growth mTORC1 [36]. Genetic alterations of mTOR signaling are found in 42% of primary and in 100% of metastatic PCa [37]. Exploration of the functional relationship between STAT3 and mTORC1 signaling in the metastatic PCa mouse model revealed a significant increase in protein levels of mTORC1 downstream substrates p-4E-BP1, 4E-BP1 and the phosphorylated ribosomal protein S6 in *Pten*^*pc−/−*^ *Stat3*^*pc−/−*^ mice compared to *Pten*^*pc−/−*^ *Stat3*^*C/*+^ mice (Fig. 2D, E), supporting the role for STAT3 as a negative regulator of mTOR signaling in PCa. We also observed an increase of *Ampkα* and the key members of the *Lkb1 (Stk11)* complex *Mo25* and *Strada* mRNA levels in *Pten*^*pc−/−*^ *Stat3*^*C/*+^ mice (Supplementary Fig. 2C).

Having identified STAT3 as a transcriptional regulator of LKB1, we evaluated whether deletion of *Lkb1 (Stk11)* in *Pten*^*pc−/−*^ mice would lead to deregulated STAT3 or mTORC1 signaling. Since the combination of *Pten* and *Lkb1 (Stk11)* knock-out with heterozygous loss of *Pten* resulted in early lethality [29], we analyzed the *Pten*^*pc*+*/−*^ *Stk11*^*−/−*^ mouse model, which showed invasive PCa and extensive lung metastases with an incidence of > 80%. *Pten*^*pc*+*/−*^ *Stk11*^*−/−*^ tumors and lung metastases had elevated S6 as well as 4E-BP1 phosphorylation levels in comparison to *Pten*^*pc*+*/−*^ suggesting that tumors with a loss of LKB1/PTEN also increased mTORC1 activity (Fig. 2F).

Interestingly, the findings of Poffenberger et al. [30] and Ollila et al. [38] demonstrated that heterozygous loss of LKB1 (Stk11) expression in T cells or stromal cells is sufficient to induce proinflammatory cytokines, including IL-6 and IL-11, while reducing AMPK family members SIK1, MARK1 and MARK4. This raises the possibility that proinflammatory cytokines such as IL-6 or IL-11 mediate induction of JAK/STAT signaling.

### STAT3 is a central regulator of mTORC1 and CREB signaling

**Fig. 3:**
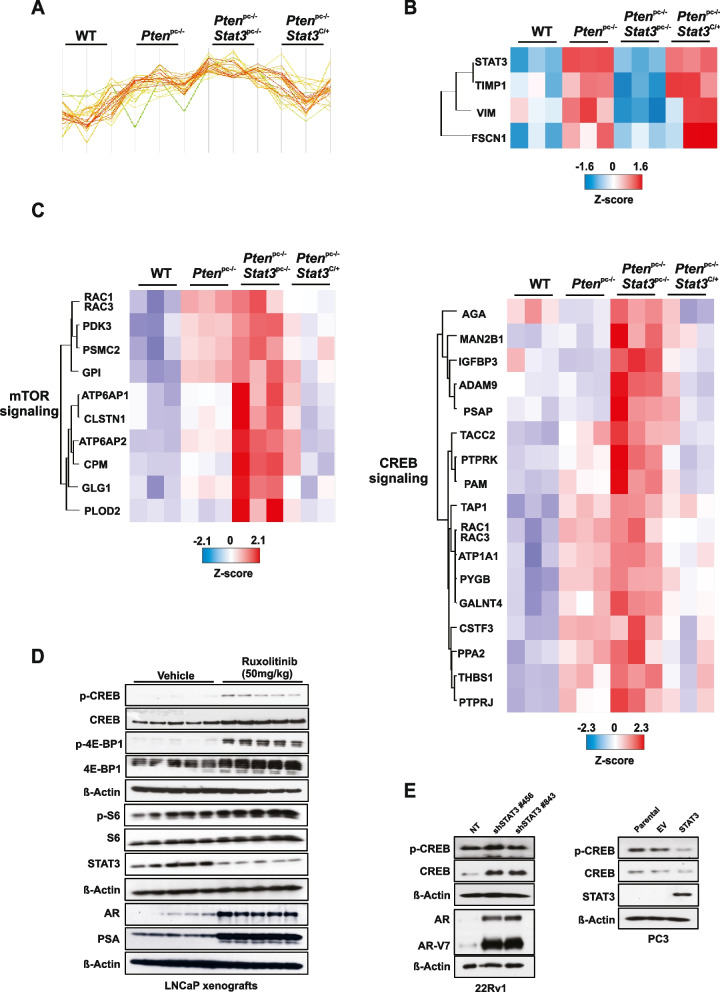
STAT3 is a central regulator of mTORC1 and CREB signaling. **A**, **B** Signature and heatmap of Stat3 and Stat3-regulated proteins in FFPE laser-microdissected prostates of 19-week-old WT, *Pten*^*pc−/−*^, *Pten*^*pc−/−*^*Stat3*^*pc−/−*^ and *Pten*^*pc−/−*^*Stat3*^*C/*+^ (*n* = 3 each) using unbiased LC–MS/MS proteomics. **C** The heatmap shows reprogramming of mTOR and CREB metabolic pathways in PCa with significant enrichment (hypergeometric test, *q*-value < 0.05). **D** Western blot analysis of p-CREB, CREB, p-4E-BP1, 4E-BP1, STAT3, p-S6, S6, AR and PSA of LNCaP xenograft tumors treated with vehicle or ruxolitinib (50 mg kg^−1^) for 22 days. β-actin serves as a loading control. **E** Western blot analysis of p-CREB, CREB, AR, AR-V7 in 22Rv1 cells transfected with non-targeting (NT) or shRNAs specific for STAT3. Western blot analysis of STA3, p-CREB and CREB in PC3 cells transfected with an empty vector (EV) or STAT3 add-back. PC3 cells lacks STAT3 expression

To identify the key oncogenic or tumor suppressor pathways involved in STAT3-dependent regulation of metastatic progression, we performed quantitative laser microdissection (LMD) of prostate epithelial cells and label‐free protein quantification (LFQ) proteome profiling on laser microdissected FFPE tumors of WT, *Pten*^*pc−/−*^, *Pten*^*pc−/−*^ *Stat3*^*pc−/−*^ and *Pten*^*pc−/−*^ *Stat3*^*C/*+^ prostate tissues. We detected 2,994 proteins that were altered between the *Stat3* deleted and constitutively active tumors (Fig. 3A). We identified very low STAT3 protein expression in *Pten*^*pc−/−*^*Stat3*^*pc−/−*^ compared to high expression in *Pten*^*pc−/−*^*Stat3*^*C/*+^ prostate samples (Fig. 3B). It has been shown that STAT3 loss lead to disruption of mitochondrial metabolism and regulated the expression of genes involved in the mitochondrial oxidative phosphorylation (OXPHOS) [39]. Conversely, Pten haploinsufficiency resulted in mitochondrial dysfunction and increased activities of I-V mitochondrial complexes [12, 40]. Our earlier [10] and current data (Supplementary Fig. 3A) showed increased levels of TCA/OXPHOS proteins in *Pten*^*pc−/−*^*Stat3*^*pc−/−*^ tumor cells compared to *Pten*^*pc−/−*^ tumors, suggesting that STAT3 is a key component of dynamic mitochondrial bioenergetics and redox regulation that enables cells to maintain homeostasis and energy metabolism under tumorigenesis and metastatic energetic stress. We next evaluated glutamine metabolism and glycolysis signaling pathways as indicators of anabolism, proliferation, and survival in cancer cells. We observed a reduction of glutamine metabolism and glycolysis protein levels in *Pten*^*pc−/−*^*Stat3*^*C/*+^ compared to *Pten*^*pc−/−*^*Stat3*^*pc−/−*^ specimens, confirming metabolic shift in biosynthetic intermediates indispensable for cancer cell energy metabolism (Supplementary Fig. 3B and C). AMPK is a master regulator of energy metabolism with one of the most widely reported functions in regulation of lipid metabolism through lipid biosynthesis enzymes, including acetyl-CoA carboxylase (ACC) and fatty acid synthase (FASN). To confirm the STAT3/LKB1/AMPK role in lipid metabolism, we performed IHC analysis of p-ACC and FASN in mouse PCa specimens and observed decrease in p-ACC and FASN in *Pten*^*pc−/−*^*Stat3*^*C/*+^ compared to *Pten*^*pc−/−*^*Stat3*^*pc−/−*^ specimens (Supplementary Fig. 3D). To explore differentially expressed tumor intrinsic signaling pathways in advanced PCa, we analyzed STAT3-dependent signaling profile at the proteome level. We observed that tissue inhibitor of metalloproteinases 1 (TIMP1), Vimentin (VIM) and actin-bundling protein fascin 1 (FSCN1) were over-expressed in the context of *Stat3* activation (Fig. 3B). TIMP1 is a known STAT3 downstream target gene [41] and other studies suggested that VIM [42] and FSCN1 [43] are regulated through STAT3 phosphorylation and could be possible STAT3 targets. Transcriptional activation by STAT3 has been shown to require the recruitment of CREB-binding protein (CBP)/p300 coactivators. In line with these results, CBP/p300 can interact with the activation domain of STAT3 to regulate transcription [44]. A recent study by Li et al*.* [20] demonstrated that CREB activity is required for mTORC1 signaling in primordial follicles. This coexisting activation of CREB and mTORC1 activity remains unclear in cancer. Here, we performed comprehensive global protein expression profiling, which showed an upregulation of mTOR and CREB signaling pathways (Fig. 3C) in *Pten*^*pc−/−*^*Stat3*^*pc−/−*^ tumor cells, suggesting a critical negative regulatory role of STAT3 for mTORC1 and CREB signaling in PCa tumorigenesis and metastatic formation. To assess the effect of STAT3 inhibition on mTOR and CREB signaling in vivo, we treated human LNCaP xenografts with JAK/STAT inhibitor ruxolitinib. Interestingly, p-CREB, CREB, p-4E-BP1, 4E-BP, p-S6 and S6 protein levels were prominently upregulated in LNCaP xenografts treated with ruxolitinib (Fig. 3D), suggesting that JAK/STAT inhibition may directly induce CREB and mTORC1 pathways. Interestingly, in LNCaP xenografts treated with ruxolitinib, AR and PSA protein levels are prominently upregulated, which suggests rising PSA and AR levels demonstrated in clinical mPCa. Accordingly, STAT3 knockdown in 22Rv1 PCa cells lead to upregulation of CREB signaling and AR expression including the AR-V7 variant (Fig. 3E) and wild-type AR (Supplementary Fig. 3E). STAT3 add-back in PC3 cells genetically lacking STAT3 led to reduced p-CREB and CREB protein levels (Fig. 3E). Taken together, these results imply that STAT3 may preferentially suppress the CREB and mTORC1 pathways and blunt the androgen response.

### A clinically relevant dose of metformin inhibits mTORC1/CREB in a manner that requires AR and STAT3 signaling

Treatment of PCa with androgen deprivation therapy (ADT) leads to a metabolic syndrome that can contribute to cancer-related morbidity and mortality. Metformin can reverse the effects of the metabolic syndrome but also was shown to have a potential antineoplastic effect in several malignancies [45].

**Fig. 4:**
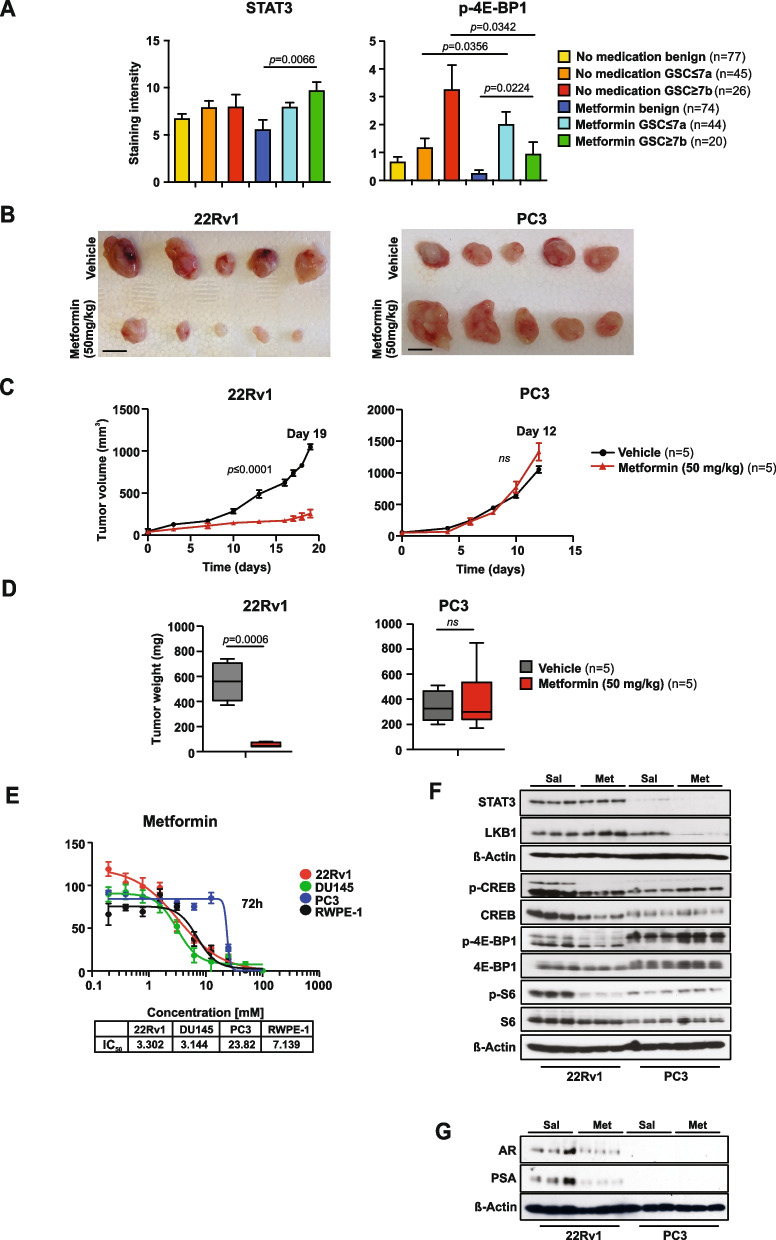
Clinically relevant dose of metformin inhibits CREB/mTORC1 in a manner that requires AR and STAT3 signaling. **A** IHC of radical prostatectomy specimens (benign and cancer core) stained with STAT3 and p-4E-BP1. TMA including benign and cancerous tissue with either metformin (*n* = 41) or patients without antidiabetic medication (*n* = 39) was employed as described previously [46]. **B** Gross anatomy assessment of representative 22Rv1 and PC3 xenograft tumors treated with vehicle or metformin (50 mg/kg; i.p.). Scale bars, 10 mm. Mean values are shown; error bars: s.d. (*n* = 5). **C** 22Rv1 and PC3 cells were implanted subcutaneously in mice and grown until tumors reached the size of approximately 100mm^3^. Xenografted mice were randomized and then received (*n* = 5 per group) vehicle or 50 mg/kg metformin i.p. daily. Mean tumor volume ± s.d. is shown. **D** Tumor weights assessment of representative 22Rv1 and PC3 xenograft tumors treated with vehicle or metformin (50 mg/kg; i.p.). Scale bars, 10 mm. Mean values are shown; error bars: s.d. (*n* = 5). **E** Comparison of IC_50_ values of metformin for human PCa cell lines (22Rv1, DU-145 and PC3) and untransformed human prostate cell line RWPE-1. **F** Western blot analysis of STAT3, LKB1, p-CREB, CREB, p-4E-BP1, 4E-BP1, p-S6 and S6 in 22Rv1 and PC3 xenograft tumors. β-actin serves as a loading control. Sal…physiological saline solution; Met…metformin. **G** Western blot analysis of AR and PSA in 22Rv1 and PC3 xenograft tumors treated with vehicle or metformin (50 mg/kg; i.p.). β-actin serves as a loading control

To investigate the clinical relevance of STAT3-mTORC1 signaling in human PCa, we performed antibody staining for STAT3 activation and the mTORC1 substrates p-4E-BP1, 4E-BP1 and p-S6 in tissue microarrays (TMA) of patients diagnosed with t2DM who underwent radical prostatectomy due to organ confined PCa as described previously [46]. Patients were treated with metformin (*n* = 41). As a control group we used patients with high HbA1c but who did not fulfill the criteria for pharmacological treatment and who underwent dietary interventions (*n* = 39). Patient characteristics of the cohort were described previously [46]. In total, 286 tissue samples from 80 patients were collected. Embedded hepatic cells as well as prostate cell lines (LNCaP and PC3) were used as controls. PCa samples were prognostically scored using the Gleason score (GSC) with good prognosis less than or equal to a score of 7a and poor prognosis greater or equal to 7b. We observed higher STAT3 and lower p-4E-BP1 protein expression levels in the metformin-administered group GSC $\ge$ 7b (*n* = 20) compared to the patient group GSC $\ge$ 7b (*n* = 26), which did not receive metformin (Fig. 4A). These data are consistent with the established role of metformin as an inhibitor of mTORC1 signaling through AMPK and tuberous sclerosis complex (TSC) [21]. The expression levels of other downstream substrates functionally controlling the mTORC1 complex such as total 4E-BP1 and phospho-S6 levels remained unchanged, suggesting that alternative oncogenic events likely contribute to the signaling effects of metformin (Supplementary Fig. 4A).

The frontline and most prescribed antidiabetic drug metformin also showed AMPK-dependent mTORC1 inhibition via TSC/RHEB and has been considered as a potential anticancer agent [47]. Metformin is still being evaluated as an adjuvant therapy in various disease settings [48]. To investigate whether the STAT3 status could directly affect metformin sensitivity through modulation of CREB and mTORC1 signaling, we analyzed the antiproliferative effects of metformin. We found that metformin treatment in a clinically relevant dose of ~ 70 µM [49, 50] decreased size, weight, and tumor volume in AR/STAT3 expressing 22Rv1 cells but was ineffective for PC3 xenografts, which lack STAT3 (Fig. 4B-D). Metformin also significantly reduced cell viability in all PCa cell lines except the PC3 cell line, which is androgen insensitive and is known to lack both, AR and STAT3 [51] (Fig. 4E). Metformin inhibits mitochondrial electron transport chain (ETC) complex I as well as other targets of mitochondrial metabolism [52]. Furthermore, it has been shown that acute and chronic low dose metformin treatment effectively impacted the cytosolic/mitochondrial redox state and inhibited mitochondrial glycerol phosphate dehydrogenase (mGPD) [53]. Since both, complex I inhibition and decreased mGPD activity could lead to NAD^+^ deficiency, we measured NAD^+^, NADH and ATP in 22Rv1 and PC3 cells in vivo using high-resolution mass spectrometry (HRMS) and observed decreased levels of NAD^+^ in PC3 cells (Supplementary Fig. 4B) consistent with an altered NAD^+^ regeneration and sensitivity leading to malignant phenotypes by promoting clonal cell growth and migration upon loss of STAT3 in triple negative breast cancer [54]. In contrast to 22Rv1, PC3 tumors treated with metformin exhibited high proliferative counts of Ki-67^+^ cells associated with an increased number of CC3^+^ apoptotic cells (Supplementary Fig. 4C). Importantly, we observed that metformin treatment suppressed mTORC1 and CREB signaling in STAT3-expressing 22Rv1 xenografts while the ability of metformin to suppress p-4E-BP1, 4E-BP1, p-S6, S6, p-CREB and CREB was greatly diminished in STAT3-deficient PC3 xenografts (Fig. 4F), underlying the mTORC1 and CREB dependency on STAT3 protein expression status. Similarly, induction of LKB1 was observed in STAT3-expressing 22Rv1 xenografts treated with metformin while blunted in PC3 STAT3-deficient tumors (Fig. 4F).

We performed a series of studies to clearly establish STAT3 as a functional mediator of metformin antitumor effect. First, we used colony formation assay with knockdown of STAT3 in 22Rv1 using different concentrations of metformin. We observed that STAT3 suppression abolished metformin tumorigenicity effect, therefore confirming STAT3-dependent effect of metformin (Supplementary Fig. 4D). Second, STAT3 knockdown indicated decreased number of apoptotic cells in metformin or rotenone treated cells, therefore, restricting the ability to inhibit mitochondrial complex I (Supplementary Fig. 4E). Moreover, STAT3 knockdown diminishes the effect of treatment with metformin in clinically relevant dose on the oxygen consumption rate (OCR) of 22Rv1 tumor cells, hence suggesting a higher protonophoric capacity and resistance to metformin treatment upon STAT3 reduction (Supplementary Fig. 4F).

We next determined whether STAT3 is critical for sustained AR signaling promoted by LKB1/AMPK. Metformin treatment repressed AR and PSA levels in 22Rv1 xenografts while PC3 tumors lacked AR and PSA protein expression (Fig. 4G). These results indicate that STAT3 and CREB signaling are critical for AR regulation and potential mPCA. Taken together, these results demonstrate that in PCa mTORC1 and CREB signaling are regulated by LKB1-AMPK in an STAT3-dependent manner and may control PCa cell growth and metastatic spread.

### STAT3 and PTEN are negatively correlated with mTORC1 in PCa

**Fig. 5:**
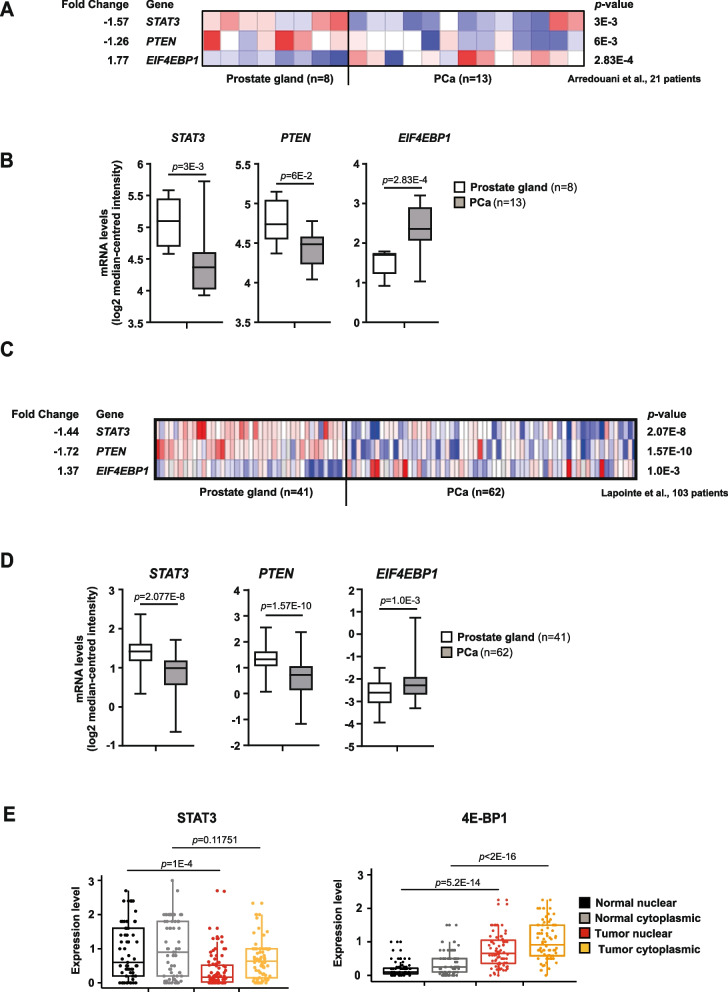
STAT3 and PTEN are negatively correlated with mTORC1 in PCa. **A** Heatmaps depicting significant downregulation of *STAT3* and *PTEN* mRNA levels and concomitant upregulation of *EIF4EBP1* mRNA expression in prostate carcinoma patient samples (*n* = 13) compared with healthy prostate gland tissues (*n* = 8). Colors were normalized to depict relative mRNA expression values (log2 median-centered intensity) within each row; dark blue represents the lowest relative expression level and dark red represents the highest relative expression level. Data were extracted from the Oncomine™ Platform [59] and from the Arredouani Prostate study [55]. **B** Gene expression levels depicting significant downregulation of *STAT3* (-1.57-fold) and *PTEN* (-1.26-fold) mRNA and concomitant upregulation of *EIF4EBP1* mRNA (1.77-fold) in prostate carcinoma patients (*n* = 13) compared with normal prostate gland samples (*n* = 8). Data (log2 median-centered intensity) were extracted from the Oncomine™ Platform from the Arredouani Prostate dataset*.* Representation: boxes as interquartile range, horizontal line as the mean, whiskers as lower and upper limits. **C** Heatmap depicting significant downregulation of *STAT3* and *PTEN* mRNA levels and concomitant upregulation of *EIF4EBP1* mRNA expression in prostate carcinoma patients compared with normal prostate gland samples (log2 median-centered intensity). Data were extracted from the Oncomine™ Platform from the Lapointe Prostate dataset*.* **D** Gene expression levels depicting significant downregulation of *STAT3* (-1.44-fold) and *PTEN* (-1.72-fold) mRNA and concomitant upregulation of *EIF4EBP1* mRNA (1.37-fold) in prostate carcinoma patients (*n* = 59–62) compared with normal prostate gland samples (*n* = 37–41). Data (log2 median-centered intensity) were extracted from the Oncomine™ Platform from the Lapointe Prostate dataset*.* Representation: boxes as interquartile range, horizontal line as the mean, whiskers as lower and upper limits. **E** Boxplots representing protein expression of STAT3 and 4E-BP1 in cytoplasmatic or nuclear stainings detected by IHC in normal-like glands or tumors in PCa patient TMAs (*n* = 83)

In our model systems, STAT3 and PTEN loss resulted in upregulation of mTORC1 as seen in primary and metastatic PCa. We explored publicly available data sets of PCa and metastatic PCa from Arredouani et al*.* [55] and Lapointe et al*.* [56]. The cohorts revealed significant mRNA downregulation of *STAT3*, *PTEN* and upregulation of *EIF4EBP1* (4E-BP1) (Fig. 5A-D). We further expanded our analysis of mRNA expression levels for STAT3, PTEN and mTORC1 substrate EIF4EBP1 using large datasets of the TCGA PCa [57] and GSE3325 (Supplementary Fig. 5A-D). Similar results confirmed clinical failure and more likely development of distant metastasis occurring in patients [58] in the presence of low PTEN and high EIF4EBP1 and EIF4E mRNA expression, which resulted in significantly prolonged metastatic free survival (Supplementary Fig. 5E). We then focused our analysis on TMA of PCa patient cohorts (*n* = 83). In line with our findings in mouse model systems and patient data sets, low STAT3 and high 4E-BP1 and p-4E-BP1 cytoplasmic and nuclear protein expression were seen in PCa (Fig. 5E, Supplementary Fig. 5F). Further research showed that increased p-4E-BP1 protein expression was present in advanced Gleason 5 carcinoma compared to Gleason 3 or 4 PCa (Supplementary Fig. 5G). In summary, our findings from PCa patient samples mimic the molecular phenotype of preclinical mouse models and engineered cell lines, which exhibited reprogramming of mTORC1 metabolic pathways.

### CREB signaling predicts ADT-resistance and metastatic progression in PCa patients

**Fig. 6:**
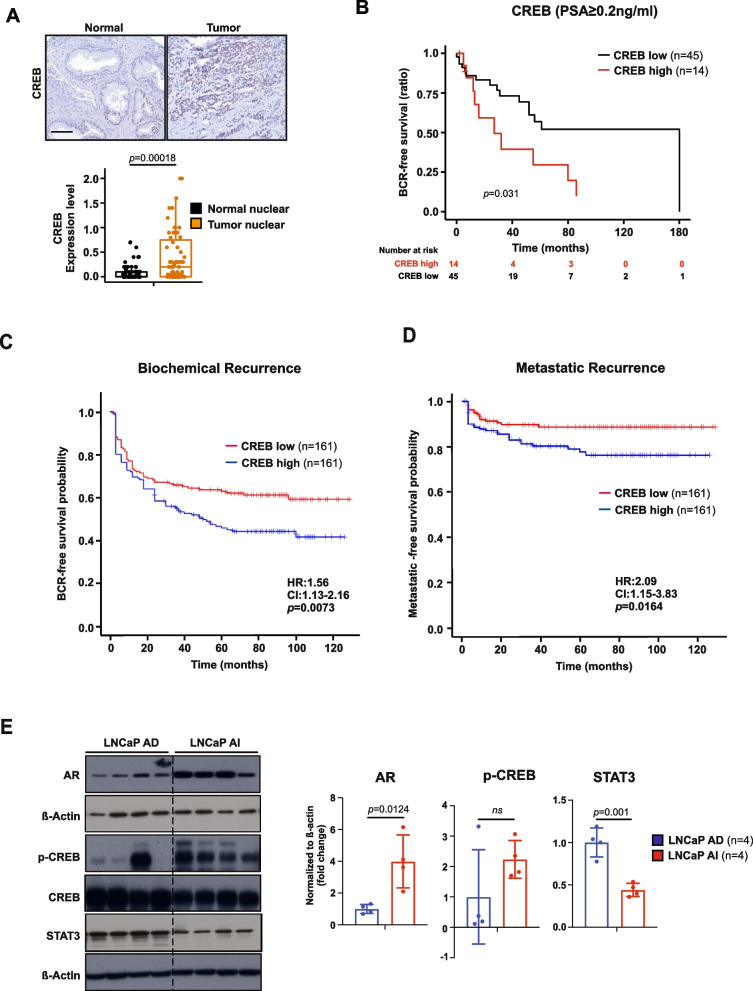
CREB signaling predicts ADT-resistance and metastatic progression in PCa patients. **A** Representative IHC images and boxplots representing protein expression of CREB in nuclear stainings detected by IHC in normal-like glands or tumor cells in PCa patient TMAs (*n* = 83). **B** Kaplan–Meier analysis of BCR-free survival ratio based on CREB protein expression in a panel of 59 PCa patients (PSA $\ge$ 0.2 ng/L). **C** Association of CREB expression at predicting time to biochemical recurrence of high/low-risk disease in the resection cohort. Reduced progression-free survival in months of the “high-risk” subgroup (blue) of 161 patients when compared with the “low-risk” subgroup (red) of 161 patients (HR = 1.56 [1.13–2.16]; *p* < 0.0073). **D** Association of CREB expression at predicting time to metastatic disease recurrence of high/low-risk disease in the resection cohort. Reduced progression-free survival in months of the “high-risk” subgroup (red) of 161 patients compared with the “low-risk” subgroup (blue) of 161 patients (HR = 2.09 [1.15–3.83]; *p* < 0.0164). HR = hazard ratio. (**E**) LNCaP xenograft model was serially passaged in castrated NSG males. WB analysis of AR, p-CREB, CREB, STAT3 protein expression in LNCAP AD/AI tumors. β-actin serves as a loading control

Considering these findings, we determined the clinical relevance of CREB signaling in patients affected by PCa by analyzing TMA of 83 patient cases. IHC analysis revealed that tumors (Fig. 6A), and PCa patients with GSC 8–10 expressed high protein levels of CREB (Supplementary Fig. 6A). Next, we determined whether CREB expression could predict worse clinical outcome. Indeed, we found that high CREB expression significantly correlates with inferior BCR-free survival in PCa patients (Fig. 6B, Supplementary Fig. 6B). On multivariable analysis, a high CREB1 gene expression pattern was associated with an increased risk of biochemical recurrence (HR = 1.56 [1.13–2.16]; *p* = 0.0703; (Fig. 6C) and metastatic recurrence (HR = 2.09 [1.15–3.83]; *p* = 0.0164; (Fig. 6D). Since CREB1 mRNA expression predicts metastatic recurrence better than biochemical recurrence this suggests that the metastatic subgroup of PCa patients with high CREB expression have a greater risk of developing metastatic disease progression. Taken together, these data suggest that targeting CREB signaling may be a promising approach for suppressing metastatic PCa in the molecular context of loss of *PTEN*. We attempted to compare transition from androgen-dependent (AD) to an androgen-independent (AI) PCa in LNCaP xenograft models during serial propagation in castrated mice [60] (Fig. 6E). We observed a clear upregulation of AR and p-CREB protein levels and STAT3 downregulation in AR-independent LNCaP xenografts linking the CREB expression to distinct tumorigenic behavior of CRPC and suggesting therapeutic regiments targeting CREB in mCRPC. In summary our findings suggest that upregulated STAT3 reduces mTORC1 and LKB1 expression. Using genetic and pharmacological approaches, we identified LKB1 as a direct STAT3 target while CREB is suppressed. PCa patients with low STAT3 and high CREB expression have a worse clinical course with a significantly increased risk of PCa with metastatic recurrence. In addition, we find that castration decreases STAT3 and LKB1 expression and increases AR and CREB levels, subsequently leading to mPCa. In conclusion, we show that STAT3 suppresses PCa growth via LKB1/mTORC and CREB is a promising downstream treatment target for lethal CRPC.

## Discussion

**Fig. 7:**
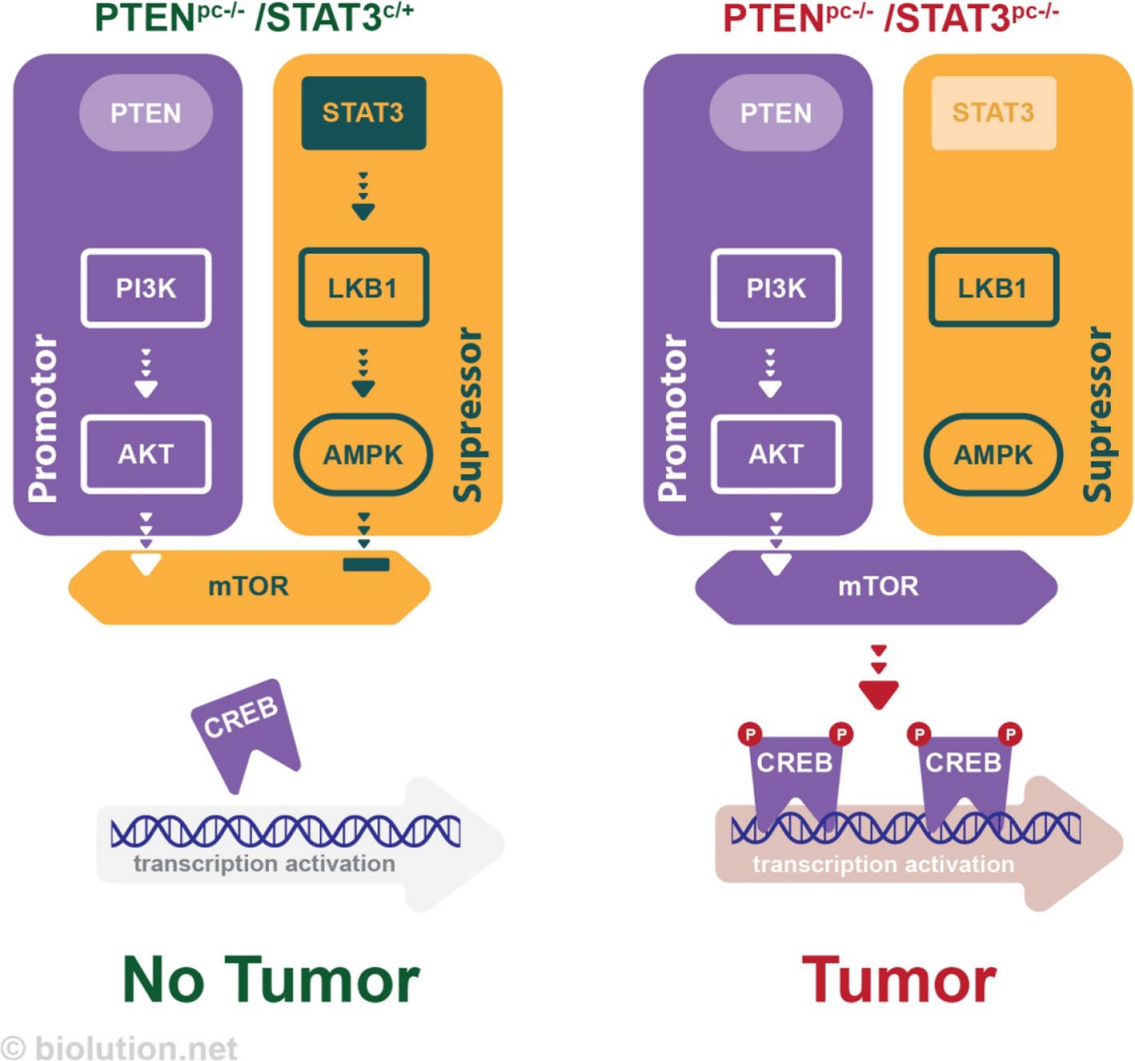
Schematic representation of the role of STAT3 in prostate cancer. In a mouse model of PCa with PTEN loss, deletion of STAT3 results in decreased levels of LKB1/pAMPK but increased activation of mTOR signaling, leading to the development of mPCa. In contrast, constitutively activation of STAT3 results in high levels of LKB1/pAMPK and decreased activity of the mTORC1/CREB pathway, thus, preventing the development of cancer in mice with PTEN loss

mPCa is a major factor in cancer-associated mortality in males, but the underlying mechanisms are not well understood. PTEN is one of the most frequently deleted genes in mPCa, and analysis of liquid biopsies from patients with mPCa has revealed frequent co-deletion of PTEN and STAT3. In a mouse model of PCa with PTEN loss, deletion of STAT3 resulted in decreased levels of LKB1/pAMPK but increased activation of mTOR signaling, leading to the development of mPCa. However, when STAT3 was constitutively activated, this resulted in high levels of LKB1/pAMPK and decreased activity of the mTORC1/CREB pathway, preventing the development of cancer in mice with PTEN loss (Fig. 7). Treatment of PCa cells with metformin, a drug used to treat t2DM, reduced mTORC1/CREB signaling and slowed the growth of androgen dependent PCa cells transplanted into mice. In addition, patients with PCa and high CREB levels had a worse clinical course, including an increased risk of recurrence and metastasis. These results suggest that STAT3 plays a role in regulating PCa growth via the LKB1/mTORC1/CREB pathway, which may be a promising target for the treatment of lethal mPCa.

While STAT3 is usually considered an oncogene, some research suggests that it may also have a tumor suppressive effect in certain cases [8, 9]. Previous work has shown that the tumor suppressive function of STAT3 is closely linked to PTEN, a tumor suppressor protein that inhibits PI3K/AKT signaling [61]. Our analysis of data from The Cancer Genome Atlas (TCGA) database suggests that decreased mRNA STAT3 levels are often associated with PTEN deletion or downregulation in metastatic tumors. In addition, cfDNA plasma samples from patients with metastatic PCa showed a high frequency of simultaneous deep deletions of PTEN and STAT3. This suggests that loss of PTEN and STAT3 is a frequent event in advanced and metastatic PCa. Therefore, the genetic relationship between STAT3 and PTEN may play an obligatory role in metastatic dissemination and tumor maintenance during therapy and progression. Using genetically engineered mouse models (GEMMs), we show that genetic inactivation of *Stat3* in *Pten*^*pc−/−*^ mice shortens survival, whereas constitutive *Stat3* activation causes tumor regression and prolongs survival beyond PTEN deficiency. Remarkably, we observed metastasis to a similar extent in Pten^pc−/−^, Stat3^pc−/−^ mice as in Pten^pc−/−^, Tp53^pc−/−^, Pten^pc−/−^, Smad4^pc−/−^ or Pten^pc−/−^, KMT2C^−/−^, GEMMs [62, 63], indicating the importance of STAT3 signaling for metastasis risk in human PCa. LKB1, a master regulator of cellular metabolism and energy stress responses, negatively regulates tumor growth and metastasis in mouse models of lung cancer and melanoma [64]. Our results also show that deregulation of LKB1/pAMPK and mTORC1 signaling is closely linked to the STAT3 status, suggesting that STAT3 signaling is a critical regulator of PCa metastasis by controlling LKB1-dependent biochemical signatures of metastatic behavior and senescence. In addition to functional inactivation of LKB1/AMPK in Pten^pc−/−^ tumors due to loss of STAT3, ChIP analysis confirmed that STAT3 binds directly to the LKB1 (STK11) promoter. This suggests that STAT3 can regulate LKB1 expression by directly binding to the promoter region of the LKB1 gene. We observed that knocking down of STAT3 leads to upregulation of mTORC1 signaling, which may be a major contributor to treatment resistance and mPCa. Using unbiased LMD (laser microdissection) and LFQ (label-free quantitation) shotgun proteome profiling, we found that tumors with *Stat3* loss that exhibited a metastatic phenotype were highly dependent on mTOR and CREB pathways. These results suggest that mTOR and CREB have important regulatory roles in the formation of metastases in PCa. Treatment with ruxolitinib, a Janus kinase inhibitor, led to increased expression of CREB and mTORC1 signaling pathways in LNCaP xenograft tumors. This may have the potential to promote the metastatic behavior and therapy resistance in PCa. CREB is a transcription factor that has been linked to the development and progression of several cancers, including PCa. It forms a complex with CREB-binding protein (CBP) and recruits the transcription machinery in the promoter region of a gene to initiate CREB-dependent gene transcription [65]. Metformin, a drug used to treat t2DM, can stimulate phosphorylation of CBP (CREB-binding protein) in a mouse model of insulin resistance [66]. Metformin has been found to decrease prostate cancer cell viability and increase apoptosis through its effects on the AR signaling pathway [67]. In mice with either STAT3-proficient or STAT3-deficient PCa, metformin had different effects on the CREB/mTORC1 pathway in the two types of tumors. In STAT3-proficient tumors, metformin had CREB/mTORC1-dependent effects, while in STAT3-deficient tumors, it appeared to have independent mechanisms of action. Furthermore, we found that STAT3 is necessary for the inhibition of AR and PSA by metformin, suggesting that targeting STAT3 and CREB could be a potential strategy for the treatment of mPCa. Additionally, high levels of CREB have been linked to tumor recurrence and metastasis in PCa patients, and patients with tumors expressing high levels of CREB tend to have shorter periods of PSA relapse. These findings suggest that targeting CREB could be a promising approach for the treatment of mPCa.

In summary our findings show that in addition to the loss of PTEN, also loss of STAT3 plays an important role in the development and progression of mPCa and that loss of STAT3 and PTEN often occurs together in advanced and mPCa. Constitutive activation of STAT3 leads to activation of LKB1/AMPK and suppresses mTORC1 pathway therefore prevents PCa formation under loss of PTEN. Furthermore, metformin, which requires STAT3 for its mTORC1-mediated inhibitory effects, may have potential as a combination therapy agent for androgen-dependent PCa. Our data also show that higher levels of CREB are frequently associated with tumor recurrence and metastasis in PCa patients, and that patients with tumors expressing high CREB levels have shorter periods of PSA relapse. These findings suggest that inhibition of mTORC1 and CREB signaling may be a potential new therapeutic modality in mPCa patients for which there are very limited treatment options.

## Methods

### Generation of transgenic mice

The prostate epithelium-specific deletion was generated by the PB-Cre crossed with *Pten*^*loxP/loxP*^ and/or Stat3^*loxP/loxP*^ conditional mice [9]. To generate constitutively activated *Stat3*, we took advantage of *Stat3*^*C/*+^ [32] and crossed them with *Pten*^*loxP/loxP*^ mice. All cohorts were in a C57BL/6 and 129/Sv mixed genetic background. Animal experiments were reviewed and approved by the Austrian ministry authorities and conducted according to relevant regulatory standards (BMWFW-66.009/0281-I/3b/2012 and BMWFW-66.009/0088-WF/V/3b/2018). The conditional tissue-specific prostate and lung metastatic samples with *Pten*^pc−/+^ (heterozygous) and/or *Lkb1* alterations were kindly provided by Arkaitz Carracedo and described previously by Hermanova et al*.* [29].

### Clinical specimens

We retrospectively analyzed PCa patients diagnosed with both t2DM and PCa who underwent an open retropubic or robotic assisted (Da Vinci) RPE. In total, 286 tissue samples from 80 patients were collected. Cylindrical samples including three cancer areas and three benign areas were re-located from formalin-fixed, paraffin-embedded tissue blocks to the TMA block. Embedded hepatic cells as well as prostate cells (LNCaP and PC3) were used as controls. A tissue micro array (TMA) was assembled using a manual tissue arrayer (Beecher Instruments). The study was approved by the Ethical Committee of the Medical University Innsbruck (study number AN2014-0145 336/4.24) and written informed consent to participate in research studies was obtained from all patients.

A second TMA PCa cohort was obtained from the Department of Pathology of the Medical University of Vienna (MUW), Vienna, Austria. The FFPE material originated from 83 primary PCa patients and seven bladder cancer patients who underwent radical prostatectomy at the General Hospital of Vienna from 1993 to 2015. Use of patient FFPE material in this study was approved by the Research Ethics Committee of the Medical University Vienna, Austria (1877/2016). Staining intensities (Int) were rated from weak (= 1) to strong (= 3) and percentage (Perc) of positive cells were evaluated. Thereby, the overall expression level (EL) was derived:

$$\text{EL} = \frac{{\text{Int}}x{\text{Perc}}}{100}$$

For group comparisons, Pearson chi-square normality test, Q–Q (quantile–quantile) plots and density plots were applied to test for normality and to visually inspect data. Levene's Test was applied to test for homogeneity of variance. Either parametric (ANOVA) or non-parametric (Kruskal–Wallis) tests were used. Pairwise comparisons were performed using Bonferroni multiple comparisons of means after ANOVA and Dunn's all pair test after Kruskal–Wallis test. Significance was defined as *p*-value ≤ 0.05. Statistical tests were performed using the R software environment with packages DescTools v.0.99.28, PMCMRplus v.1.4.1 and nortest v.1.0–4. Plots were generated with ggpubr_0.2 and ggplot2_3.3.0. Data were processed using tidyverse v.1.2.1.

### Plasma samples

The study was approved by the Ethics Committee of the Medical University of Graz (approval number 21–228 ex 09/10), conducted according to the Declaration of Helsinki, and written informed consent was obtained from all patients. At the time of the first blood collection, 6 (14.0%) patients had castration-sensitive PC (CSPC) and 37 (86.0%) patients had castration-resistant PC (CRPC). 95 plasma samples derived from 43 patients with metastatic PCa [68]. The majority of cases (35/43; 81.4%) displayed typical high-grade prostate adenocarcinoma features, five cases (11.7%) were poorly differentiated prostate cancers, one case each (2.3%) had undifferentiated or glandular histology and in one case (2.3%) the histology could not be obtained. No case showed neuroendocrine differentiation or exhibited small-cell neuroendocrine features. In the following are detailed histories of the patients with serial plasma DNA analyses.

### Immunohistochemistry and histological analysis

Immunohistochemistry and haematoxilin/eosin staining was performed with formalin-fixed paraffin-embedded (FFPE) tissue using standard protocols using consecutive sections. The following antibodies were used for immunohistochemistry: STAT3 (1:100 dilution; CST, #9139), LKB1 (1:100 dilution; Abcam; ab15095), p-AMPK 1:100 dilution; CST, #2535), p-4E-BP1 1:100 dilution; CST, #2855), 4E-BP1 1:100 dilution; CST, #9644), p-S6 1:100 dilution; CST, #2211), PTEN 1:100 dilution; CST, #9188), Ki-67 (1:1,000 dilution; Novocastra; NCL-KI-67-P) and Cleaved Caspase 3 (1:200 dilution; Cell Signaling, #9661). p-CREB 1:100 dilution; CST, #9198), CREB 1:100 dilution; CST, #9197).

IHC was performed on a Discovery-XT staining device (Ventana). The following antibodies were used: AMACR/p63 immunohistochemistry (IHC) double staining (Monoclonal Rabbit Anti-Human AMACR, clone 13H4, Dako, Code M361601-2, 1:100, CC1 and Ventana Anti-p63 (4A4) Mouse Anti-Human Monoclonal, Catalog Number: 790–4509, BM, CC1).

For quantifying expression levels an established semi-quantitative “quick score” system combining the proportion of positive cells and the average staining intensity based on the method first described by Detre et al*.* [69] was used. Briefly, quick score categories were based on both the proportion (denoted category A) and intensity (denoted category B) of positively stained cells. The proportion of positive cells (category A) was stratified into 4 groups (0: negative, 1: ≤ 30%, 2: 30–60%, 3: ≥ 60%). Average staining intensity (category B) corresponding to the presence of negative, weak, intermediate, and strong staining was given a score from 0 to 3, respectively. An average multiplicative quick score (category A × category B) for each TMA tissue core was subsequently obtained.

For electron microscopy, mouse prostate tissue was cut into 2-mm pieces and fixed in 1.6% glutaraldehyde overnight. Photographs were taken at a 4,000 × magnification using a transmission electron microscope.

All images were taken with a Zeiss AxioImager Z1, and quantification was performed with HistoQuest and StrataQuest (TissueGnostics GmbH, Vienna, Austria, www.tissuegnostics.com) as described in detail in Schlederer et al. [70]. In brief, haematoxylin staining was used for cell identification. The range of intensities of the master marker (haematoxylin) and the immunohistochemical stainings were set by autodetection of the software. All images were analyzed with the identical settings after adjustments. The results are visualized in dot plot scattergrams and/or histograms. Cut-offs (to differentiate between positive and negative cells) and gates (to accentuate between cell populations) were set in the dot blots. For statistical analysis, the raw data were imported into GraphPad Prism 6 (GraphPad Software), analyzed for significance, and processed for data output. All images were taken with the same exposure time, signal amplification and objectives.

### Western blot analysis

For protein expression analysis by western blot, frozen tissue samples and cell lysates were prepared as described [9]. Blots were blocked with 5% BSA or 5% non-fat dry milk in 1 × TBS/0.1% Tween-20 for 1 h and incubated with the primary antibody overnight at 4°C. Primary antibodies were reactive to STAT3 (1:1,000 dilution; CST #9139), LKB1 (1:1,000 dilution; CST #3047), p-4E-BP1 (1:1,000 dilution; CST #9451), 4E-BP1 (1:2,000 dilution; CST #9644), p-S6 (1:1,000 dilution; CST #4858), S6 (1:2,000 dilution; CST #2217), p-CREB (1:1,000 dilution; CST #9198), CREB (1:1,000 dilution; CST #9104), AR (1:1,000 dilution; CST #5153), PSA (1:1,000 dilution; CST #5365).

### RNA and qRT–PCR

Total RNA was isolated using Trizol (Invitrogen) according to the manufacturer’s instructions. For quantitative reverse transcription PCR (qRT–PCR) analysis, 1 μg of total RNA was reverse transcribed to cDNA using the Transcriptor First-Strand cDNA Synthesis kit (Fermentas). qRT–PCR was performed in triplicate with aa MxPro3000 and SYBR GreenERqPCR mix (Invitrogen). For qPCR analysis CFX96 Real-Time PCR Detection System (BioRad, Hercules, CA, USA) was employed using RNA expression of target genes relative to β-actin was quantified by 2ΔΔCT method. The relative amount of specific mRNA was normalized to β-actin in each sample. Primer pairs are listed in Supplementary Table 1.

### Cell culture

Primary WT and *Stat3*-null MEF were isolated by trypsin treatment of individual littermate E13.5 embryos from a cross of *Stat3*^+*/−*^ heterozygous mice. *Stat3*^+*/−*^ mice were generated from conditional *Stat3*^+*/fl*^ mice [71] by deletion of the conditional allele in vivo using *Mox2-Cre*. Cells were amplified and used in experiments starting at passage 2. *Stat3*^*−/−*^ MEFs were grown in DMEM supplemented with 10% FBS, 2 mM L-Glutamine, 0.1 mM NEAA, 20 mM HEPES and pen/strep using standard techniques. For in vitro cultures LNCaP, RWPE-1 and PC3 were cultured under standard conditions.

Short hairpin‐mediated knockdown was performed as previously described by Eberl et al. [72]. For the knockdown of *STAT3* in 22Rv1 and LNCaP cells, the following short hairpin RNA (shRNA) constructs from the Mission TRC shRNA library (Sigma) were used: scrambled control shRNA (SHC002), shSTAT3#456 (TRCN0000071456), shSTAT3#843 (TRCN000002084310.1002/ijc.31724). Transduced cells were selected for puromycin resistance, and the knockdown was verified via WB. The PC3 cells transfected with pcDNA3-TOPO-STAT3-V5 or empty vector (pcDNA3-TOPO, pc3.1) were described previously by Pencik et al. [9].

### IC50

5,000 cells were plated in 96 well flat bottom plates and treated in triplicates with DMSO (negative control), Bortezomib (10 µM; positive control), or compounds of interest. Cell viability was assessed after 72 h using the CellTiter Blue assay (Promega, Madison, WI, USA) according to the manufacturer's protocol. IC_50_ values were determined using GraphPad Prism 9 by non-linear regression.

### Colony formation assay

1 × 10^3^ cells for each of the three biological replicates were seeded in each well of a 6-well plate. After initial treatment with metformin (0 mM (1% PBS solvent control), 0.005 mM, 0.05 mM, 0.5 mM), the treatment was refreshed by media change every 48 h. Ten days after initial plating the colonies were fixed with 100% methanol and stained with 0.5% crystal violet (Sigma #C6158). The stained colonies were counted with the ImageJ software.

### Apoptosis assay

Cell apoptosis was assessed via Annexin V and DAPI staining. In a 12-well plate 2 × 10^5^ cells per well were seeded for each of the three biological replicates. On the next day, the cells were treated with metformin (0 mM (1% PBS solvent control), 0.5 mM) or rotenone (0 µM (0.1% DMSO solvent control), 0.5 µM) for 24 h each. The cells were then harvested and resuspended in Annexin V Ready Flow (Thermofisher #R37174) according to the company's instructions. The cells were counterstained with 1 µg/ml DAPI. The staining was assessed via FACS analysis using the FITC channel for the Annexin V signal and the Pacific Blue channel for the DAPI signal. The analysis was performed via FlowJo V10 software.

### Seahorse assay

22RV1 cells were adjusted to a density of 2.25 × 10^4^ cells per 180 µl/well 24 h prior to measurement with the respective metformin concentrations (50 µM, 5 mM). On the day of the assay, media were replaced with Agilent Seahorse XF RPMI medium (pH: 7.4, 5 mM HEPES). The Seahorse assay medium was supplemented with 1 mM pyruvate, 2 mM glutamine, 10 mM glucose, and metformin. Cells were washed once before being equilibrated in a non-CO_2_ incubator. A Mito Stress test was performed as previously described [73]. The drugs were used in the following final concentrations: oligomycin, 1.5 μM; carbonyl cyanide-p-trifluoromethoxyphenylhydrazone (FCCP), 2 μM; rotenone + antimycin A, 1 μM. Total protein was used to normalize cellular input. Cells were seeded in 6–8 technical replicates and the shSTAT3 was performed with two independent constructs.

### Xenograft models in NSG mice

LNCaP cells were grown in RPMI with 10% FBS, 1% Pen/Strep. Cells were split 24 h before harvesting cells were detached with trypsin, washed twice in PBS and counted. Cells were suspended in PBS at 2 × 10^6^, mixed 1:1 with Matrigel (Corning), and kept on ice until injection. Nine weeks old NSG mice received sub-cutaneous injections of 200 μL tumor matrigel mix, so that each mouse received 1 × 10^6^ cells. On day 2 mice received either oral gavage containing ruxolitinib dissolved in a PBS/DMSO solution (20% DMSO), at 50 mg/kg; or a control PBS/DMSO (ruxolitinib *n* = 5; control *n* = 5). Mice were treated every subsequent day for a total of 10 days. Mice were monitored for tumor development and sacrificed 24 days into the experiment when one of the tumors had exceeded the size limit of 1.2 cm in diameter. Tumors were dissected and weighed, then fixed in formalin (10%) for further analysis.

Cells (PC3 or 22Rv1) were harvested using trypsin, washed twice in PBS and counted. Cells were suspended in PBS at 2 × 10^6^, mixed 1:1 with Matrigel (Corning), and kept on ice until injection. Nine-week-old NSG mice received subcutaneous injections of 200 µL tumor matrigel mix, so that each mouse received 1 × 10^6^ cells per flank. Mice were monitored for tumor development; once the tumors reached 100–150 mm^3^ in size (approx. three weeks after inoculation), mice were treated with vehicle (0.9% saline) or metformin (Sigma, PHR1084) reconstituted in 0.9% saline was administered via intraperitoneal injection once daily for 12 (PC3) and 19 (22Rv1) consecutive days, respectively. Tumors were dissected and weighed, then fixed in formalin (10%) for further analysis.

### Establishing serially transplantable AD and or AI PCa xenografts

AD (i.e. androgen-sensitive) xenograft tumors, LNCaP, were routinely maintained in intact immunodeficient NSG mice^.^ To establish the CR lines, parental AD tumor cells were purified, mixed with matrigel, injected subcutaneously and serially passaged in surgically castrated immunodeficient mice. The complete method is described by Li et al. [60].

### High-resolution mass spectrometry (HRMS)

The xenografts were extracted in 1 mL cold methanol. The extracts were dried in a vacuum concentrator and reconstituted in methanol to concentrations normalized to 40 mg xenograft tissue/100 µL methanol. The samples were analyzed with liquid chromatography electrospray-time-of-flight mass spectrometer (LC-ESI-ToF–MS) (maXis impact, Bruker Daltonics, Bremen, Germany). The mass spectrometer was operated with a capillary voltage of ± 4 kV and a plate offset voltage of ± 500 V. Nitrogen gas (200°C) administered at 8 L/min was used as desolvation gas. The nebulizer pressure was 2 bar. The digitizer sample rate was set at 4 GHz and profile spectra were collected at a rate of 1 Hz. NAD^+^ was measured in positive mode and ATP and NADH were measured in negative mode. For the positive mode analysis, 10 µL reconstituted extract was injected onto a Waters Atlantis HILIC Silica column (3 µm, 2.1 × 150 mm) (Waters, Milford, MA) (30°C, flow rate was 0.25 mL/min). The aqueous mobile phase A consisted of 10 mmol/L ammonium formate (Sigma-Aldrich) with 0.1% (v:v) formic acid (Fisher chemicals, Hampton, NH). Mobile phase B consisted of acetonitrile (VWR Chemicals) with 0.1% formic acid (v:v). The following gradient, expressed as percentage of mobile phase B, was used: 0 min 95%, 0.5 min 95%, 10.5 min 40%, 15 min 40%, 17 min 95%, and 32 min 95%. For the negative mode, 10 µL reconstituted extract was injected onto Waters Xbridge BEH amide column (3.5 µm, 4.6 × 100 mm) (30°C, flow rate was 0.25 mL/min). The aqueous mobile phase A consisted of 95:5 (v:v) water:acetonitrile supplemented with 20 mmol/L ammonium acetate and ammonium hydroxide (Sigma-Aldrich). Mobile phase B was 100% acetonitrile. The following gradient program, expressed as percentage of mobile phase B, was used: 0 min 85%, 3 min 30%, 12 min 2%, 15 min 2%, 16 min 85%, and 23 min 85%.

### Chromatin immunoprecipitation assays (ChIP)

Soluble chromatin preparation and ChIP assays were carried out as described previously [74] with some modifications. In short, cells were crosslinked with 1% v/v formaldehyde for 10 min at room temperature and the crosslink was stopped by the addition of glycine to a final concentration of 125 mM for 5 min while shaking. Chromatin was sonicated using a Twin Bioruptor (Diagenode) 30 s on/off for 15 cycles at 4°C. Two hundred microgram of chromatin was used for IP with 10 μL of STAT3 (1:50, Cat#12,640, Cell Signaling) and 4 μg of IgG (1:250, Cat#10500C, Thermo Fisher Scientific) antibodies and incubated overnight. Protein–antibody complexes were bound to magnetic protein G beads (Life Technologies) for 4–5 h and washed with standard IP wash buffers for 10 min at 4°C. The crosslink was reversed by addition of 0.05 volume of 4 M NaCl overnight at 65°C. After proteinase K digestion, DNA was recovered by phenol–chloroform–isoamylalcohol extraction and dissolved in 200 μL H_2_O. Real‐time PCR of diluted ChIP DNA and corresponding input DNA was performed on ViiA 7 Real‐Time PCR system (Thermo Fisher Scientific). Primer sequences used for ChIP are listed in Supplementary Table 1. Known STAT3 binding sites in BATF and JUNB promoters described in Tripathi et al. [75]*.* were chosen as positive controls and confirmed by extraction of corresponding peaks from ENCODE STAT3 ChIP‐Seq HeLa‐S3 data (ENCSR000EDC) with UCSC Genome Browser (http://genome.ucsc.edu). For the generation of STK11, a STAT3 binding site in the promoter region of STK11 detected by ENCODE STAT3 ChIP‐Seq HeLa‐S3 and STK11 used in Linher-Meville et al*.* [35]. Primer pairs were created with Primer3web v4.1.0 software [76].

### Liquid chromatography tandem mass spectrometry (LC‐MS/MS)

For LC–MS/MS proteomics, FFPE tumor material was used from 19-week-old WT, *Pten*^*pc-/-*^, *Pten*^*pc−/−*^ *Stat3*^*pc−/−*^ and *Pten*^*pc−/−*^*Stat3*^*C/*+^ mice (*n* = 3). Blocks were sliced into 3‐μm‐thick sections, mounted on slides, and stained with hematoxylin and eosin. Tumor areas were marked by a pathologist. To obtain proteomic profiles solely from the tumor, stroma and immune cells were excluded from dissection. One hundred nanoliter (10 mm^2^ of 10 μm slides) of FFPE material per sample was used for analysis. Lysis of microdissected tissue was carried out in 50% trifluoroethanol (TFE), 5 mM dithiothreitol (DTT), 25 mM ammonium bicarbonate (ABC) at 99°C for 45 min. followed by 5‐min. sonication (Bioruptor, Diagenode). After centrifugation at 16,000 g for 10 min., the cleared protein lysate was alkylated with 20 mM iodoacetamide for 30 min. at room temperature. Upon vacuum centrifugation, digestion was carried out in 5% TFE, 50 mM ABC to which 0.15 μg of LysC and 0.15 μg of trypsin were added for digestion overnight at 37°C. The following day, digestion was arrested by adding trifluoroacetic acid (TFA) to 1% and the digestion buffer removed by vacuum centrifugation. Peptides were suspended in 2% acetonitrile and 0.1% TFA and purified on C18 StageTips. Finally, purified peptides were resolved in 2% acetonitrile and 0.1% TFA, and the entire sample was injected for MS analysis in a single‐shot measurement. Protocols were adapted from Roulhac et al. [77]*.* and Wang et al*.* [78]*.*

LC‐MS/MS analysis was performed on an EASY‐nLC 1000 system (Thermo Fisher Scientific) coupled online to a Q Exactive HF mass spectrometer (Thermo Fisher Scientific) with a nanoelectrospray ion source (Thermo Fisher Scientific). Peptides were loaded in buffer A (0.1% formic acid) into a 50‐cm‐long, 75‐μm inner diameter column in house packed with ReproSil‐Pur C18‐AQ 1.9 μm resin (Dr. Maisch HPLC GmbH) and separated over a 270‐min gradient of 2–60% buffer B (80% acetonitrile, 0.1% formic acid) at a 250 nL/min flow rate. The Q Exactive HF operated in a data‐dependent mode with full MS scans (range 300–1,650 m/*z*, resolution 60,000 at 200 m/*z*, maximum injection time 20 ms, AGC target value 3e6) followed by high‐energy collisional dissociation (HCD) fragmentation of the five most abundant ions with charge ≥ 2 (isolation window 1.4 m/*z*, resolution 15,000 at 200 m/*z*, maximum injection time 120 ms, AGC target value 1e5). Dynamic exclusion was set to 20 s to avoid repeated sequencing. Data were acquired with the Xcalibur software (Thermo Scientific). Xcalibur raw files were processed using the MaxQuant software v.1.5.5.2 (Cox & Mann, 2008) [79], employing the integrated Andromeda search engine (Cox et al., 2011) [80] to identify peptides and proteins with a false discovery rate of < 1%. Searches were performed against the Mouse UniProt database (August 2015), with the enzyme specificity set as “Trypsin/P” and 7 as the minimum length required for peptide identification. N‐terminal protein acetylation and methionine oxidation were set as variable modifications, while cysteine carbamidomethylation was set as a fixed modification. Matching between runs was enabled to transfer identifications across runs, based on mass and normalized retention times, with a matching time window of 0.7 min. Label‐free protein quantification (LFQ) was performed with the MaxLFQ algorithm [81] where a minimum peptide ratio count of 1 was required for quantification. Data pre‐processing was conducted with Perseus software, v.1.5.5.5 for mouse data. Data were filtered by removing proteins only identified by site, reverse peptides, and potential contaminants. After log2 transformation, biological replicates were grouped. Label‐free protein quantification intensities were filtered for valid values with a minimum of 70% valid values per group, after which missing data points were replaced by imputation. The resulting data sets were exported for further statistical analyses using R. Filtered, normalized, and log2‐transformed data were imported, and PCA and unsupervised hierarchical clustering were performed. Plots were generated with ggplot2 v.3.1.1. (Wickham, 2016) [82], gplots v.3.0.1.1 and EnhancedVolcano v.1.0.1 R packages.

### Statistical analyses

Data were analyzed using GraphPad Prism 6 software. For comparing two groups unpaired Student's *t*-test and for comparing more than two groups Tukey’s post hoc test was used. Fisher’s exact test was employed when differences in distributions within groups were monitored. For Kaplan–Meier analysis and log-rank statistical evaluation of time to BCR, as well as evaluation of prognostic power in univariate and multivariate analysis, we used the IBM SPSS version 22 program. Survival analyses were performed using R packages survival_3.2–7 and survminer v0.4.6 Univariate Cox proportional hazards (PH) models were fitted for candidate genes. Log-rank tests for Cox PH significant genes with adj. *p*-value ≤ 0.01 were performed after a median split of samples by gene expression. All statistical tests were considered.

### Software environment

Data acquisition, differential expression analyses, gene set testing and statistical analyses on RNA-seq data and tissue micro arrays were performed using the R software environment (https://cran.r-project.org/) with R versions 3.6.1. and 3.6.3 and packages as mentioned in the respective sections.

### Data acquisition

TCGA PRAD RNA-seq harmonized data (https://portal.gdc.cancer.gov/projects/TCGA-PRAD) (The Cancer Genome Atlas Research Network, 2017) were acquired by TCGAbiolinks v.2.10.5 [83]. Data were normalized and transformed with edgeR v3.24.3. Normalized log2 counts of MSKCC PCa mRNA data (GEO: GSE21032) were derived from http://cbio.mskcc.org/cancergenomics/prostate/data/.

### Gene set testing

From TCGA PRAD data, a low-STAT3 (*n* = 100) and a high-STAT3 (*n* = 100) subset were selected, consisting of the 0.2^nd^ and the 1- 0.8^th^ quantile of overall STAT3 expression (cpm), respectively. After differential expression analysis (min. log-fold change = 0, max. *p*-value = 1) between low-STAT3 and high-STAT3 using limma v.3.40.6, genes were ranked by their moderated t-statistic. Gene set testing with fgsea_1.10.1) was performed on ranked genes with 10.000 permutations, a minimum gene set size = 1 and an infinite maximum gene set size. *P*-values were adjusted by Benjamini–Hochberg correction. Significance was defined by an adj. *p*-value ≤ 0.05. Molecular Signatures Database (MSigDB) C2 gene sets were acquired by msigdbr_7.0.1 package.

### Oncomine database analysis

Gene expression analysis of *STAT3, PTEN* and *EIF4EBP1* was performed in various prostate cancer datasets representing normal, tumor or metastatic samples deposited in the Oncomine Research Premium Edition database (Thermo Fisher, Ann Arbor, MI). For the analysis, the *P* value threshold was set to 0.05, the fold‐change threshold was set to 1.5 and the gene rank threshold was set to “all.”

### Biochemical and metastatic recurrence analysis in PCa patients

The Walker et al*.* [84] cohort consists of 322 FFPE prostatectomy samples. The Jain et al. [58] cohort consists of 248 FFPE biopsy samples. The Walker et al*.* cohort was dichotomised by median CREB1 expression. Cox proportional hazards regression method was used to estimate the univariate hazard ratio (HR) of the CREB1 expression categories.

### Supplementary Information

**Additional file 1:** **Supplementary Fig. 1.** (A) Circos plot of STAT3 and PTEN deletions: inner circle TCGA (*n*=492) and outer circle plasma-DNA samples from advanced PCa patients (*n*=43). (B) Gross anatomy of representative prostates isolated at 19 weeks of age from WT, *Pten*^*pc−/−*^, *Pten*^*pc−/−*^Stat3^*pc−/−*^ and*Pten*^*pc−/−*^*Stat3*^*C/+*^ mice. Scale bars, 10 mm. (C) Transmission electron microscopy (TEM) pictures of mitochondria from WT, *Pten*^*pc−/−*^, *Pten*^*pc−/−*^*Stat3*^*pc−/−*^ and* Pten*^*pc−/−*^*Stat3*^*C/+*^ prostates, 19 weeks of age mice. Red arrowheads show defected mitochondrial shape. Stat3 activation in *Pten*^*pc−/−*^*Stat3*^*C/+*^ cells reversed defected mitochondrial phenotype. **Supplementary Fig. 2.** (A) Western blot analysis of STAT3, LKB1, p-4E-BP1, 4E-BP1, p-S6 and S6 expression in WT and Stat3KO MEFs. β-actin servesas a loading control. (B) qRT–PCR analysis of *STAT3* and *STK11 *transcript levels in WT and Stat3KO MEFs (*n*=3 each). (C) qRT–PCR analysis of *Stk11, Mo25, Strada *and* Ampkα *mRNA expression in prostates of 19-week-old WT, *Pten*^*pc−/−*^, *Pten*^*pc−/−*^*Stat3*^*pc−/−*^ and *Pten*^*pc−/−*^*Stat3*^*C/+*^ (*n*=3 each). Data were analyzed by Student’s *t*-test and are shown as mean ± s.d. **Supplementary Fig. 3.** (A) The heatmap of murine PCa proteomics with significant enrichment of genes involved in regulation of oxidative phosphorylation. 19-week-old WT,* Pten*^*pc−/−*^, *Pten*^*pc−/−*^*Stat3*^*pc−/−*^and *Pten*^*pc−/−*^*Stat3*^*C/+*^ mice (*n*=3). (B, C) Heatmap of murine PCa proteomics with significant enrichment of genes involved in regulation of glutamine metabolism and glycolysis. 19-week-old WT,* Pten*^pc−/−^, *Pten*^pc−/−^*Stat3*^pc−/−^ and *Pten*^*pc−/−*^*Stat3*^*C/+*^ mice (*n*=3). (D) IHC analysis of p-ACC and FASN inprostates from 19-week-old WT, *Pten*^*pc−/−*^, *Pten*^*pc−/−*^ *Stat3*^*pc−/−*^ and *Pten*^*pc−/−*^*Stat3*^*C/+*^ mice. Scale bars, 100 μm. Bar graphs indicate percentage of cellspositive for p-ACC and FASN in prostates of 19-week-old WT, *Pten*^*pc−/−*^, *Pten*^*pc−/−*^*Stat3*^*pc−/−*^ and *Pten*^*pc−/−*^*Stat3*^*C/+*^. Protein levels quantification was done with HistoQuest software (n≥3). (E) Western blot analysis of AR and STAT3 in LNCaP cells transfected with non-targeting (NT) or shRNAs specific for STAT3. **Supplementary Fig. 4.** (A) IHC and representative images analysis of radical prostatectomy specimens (benign and cancer core) of 80 PCa patients stained with 4E-BP1 and p-S6. 41 used metformin and 39 PCa patients with nopharmacological treatment were included as a control group. (B) HRSM measurement of NAD^+^, NADH and ATP in22Rv1 and PC3 tumors (*n*=5). (C) H&E and IHC stainings of Ki-67, Cleaved Caspase 3 (CC3) and STAT3 expression invehicle versus metformin-treated xenografted tumors (*n*=5), scale bar 50 µm. (D) Colony forming units of 22Rv1 cells (NT (non-targeting), shSTAT3 #446, shSTAT3 #843) using different concentrations of metformin (0 mM (1% PBS solventcontrol), 0.005 mM, 0.05 mM, 0.5 mM) and quantification of formed colonies (*n*=3 each). (E) Assessment of apoptosis in 22Rv1 cells (NT (non-targeting), shSTAT3 #446, shSTAT3 #843) after metformin (0 mM (1% PBS solventcontrol), 0.005 mM, 0.05 mM, 0.5 mM) or rotenone (0 µM (0.1% DMSO solvent control), 0.5 µM) treatment (*n*=3 each). (F) Oxygenconsumption rate (OCR) curves of 22Rv1 cells (NT (non-targeting), shSTAT3 #446, shSTAT3 #843) after metformin treatment (50 µM, 5 mM) are shown. Injections of oligomycin (Injection 1), FCCP (Injection 2), and rotenone and antimycin A (R/A; Injection 3) are indicated by arrows. The resulting maximal respiration is shown on the right. **Supplementary Fig. 5.** (A) TCGA gene expression data for STAT3, PTEN and EIF4EBP1 were obtained from cBioPortal. TPM expression data were processed by adding a constant of 1.1 and subsequently log2 transformation. Boxplots were generated to visualize the difference inexpression between normal and cancer samples in TCGA. *P*-values are provided which were output using the unpaired Student's *t*-test to test the significance ofthe difference in mean expression between groups [57]. (B) Scatterplots were generated to visualize the relationship inexpression between each pair of genes (PTEN-STAT3, PTEN-EIF4EBP1 and STAT3-EIF4EBP1) in PCa samples in TCGA. The Pearson correlation ­­(*R*) and the *p*-value of this correlation are indicated on each plot. For these correlation analyses, the median summarizedexpression was used. (C) GSE3325 was processed by downloading the raw data as .CEL files from GeneExpression Omnibus (GEO) and performing RMA normalization resulting in log2-RMA normalized expression values. Boxplots are provided using the median summarized expression and at each individual probe set level (in case that is of interest). (D) Scatterplots weregenerated to visualized the relationship in expression between each pair ofgenes (PTEN-STAT3, PTEN-EIF4EBP1 and STAT3-EIF4EBP1) in prostate cancer samplesin GSE3325. The Pearson correlation ­­(*R*) and the* p*-value of this correlation are indicated on each plot. For these correlation analyses, the median summarized expression was used. (E) *PTEN, EIF4EBP1, EIF4E mRNA* levels of 247 patients with localized/locally advanced PCa commencing radical radiotherapy (with/without ADT) analyzed by RNA-Seq (Jain et al., 2018) [58]. Groups were generated by a median split. Significance was estimated by log-rank test (95% confidence interval) and *p*-value was adjusted with Benjamini-Hochberg method. + = censored. (F) Boxplot representing protein expression of p-4E-BP in cytoplasmatic or nuclear stainings detected by IHC in normal-like glands or tumors in PCa patient TMAs (*n*=83). (G) Evaluation and Gleason pattern annotations of nuclear and cytoplasmic IHC stainings of p4E-BP1 in PCa patient TMAs (*n*=83). Boxplots show median, 1st and 3rd quartiles, and whiskers extend to ± 1.5 interquartile range. Kruskal-Wallisand Dunn’s all pair tests were performed to assess significance (95% confidence interval). **Supplementary Fig. 6.** (A) Boxplot representing CREB nuclear protein expression stainings and representative images detected by IHC in PCa patient TMAs (*n*=83), (low GSS≤7 and high GSC=8-10). (B) Kaplan–Meier analysis of BCR-free survival ratio based on CREB protein expression in a panel of 59 PCa patients (PSA≥0.4ng/mL). **Supplementary Table 1**.

## Data Availability

Not applicable.

## Abbreviations

4E-BP1: Eukaryotic translation initiation factor 4E-binding protein 1
ACC: Acetyl-CoA carboxylase
AD: Androgen-dependent
AI: Androgen-independent
AKT: Protein kinase B
AMP: Adenosine monophosphate
AMPK: 5' Adenosine monophosphate-activated protein kinase
AR: Androgen receptor
ATP: Adenosine triphosphate
BSA: Bovine serum albumin
CBP: CREB-binding protein
ChIP: Chromatin immunoprecipitation assays
CREB: CAMP response element-binding protein
CRPC: Castration-resistant prostate cancer
CSPC: Castration-sensitive prostate cancer
DAPI: 4′,6-Diamidino-2-phenylindole
DMSO: Dimethyl sulfoxide
FASN: Fatty acid synthase
FBS: Fetal bovine serum
FCCP: Carbonyl cyanide-p-trifluoromethoxyphenylhydrazone
FFPE: Formalin-fixed paraffin-embedded
FITC: Fluorescein isothiocyanate
FSCN1: Fascin actin-bundling protein 1
GSC: Gleason score
HEPES: 4-(2-Hydroxyethyl)-1-piperazineethanesulfonic acid
HRMS: High-resolution mass spectrometry
JAK: Janus kinase
LC‐MS/MS: Liquid chromatography tandem mass spectrometry
LKB1: Liver kinase B1 also known as serine/threonine kinase 11 (STK11)
MEF: Mouse embryonic fibroblasts
mGPD: Mitochondrial glycerol phosphate dehydrogenase
mPC: Metastatic prostate cancer
mTOR: Mammalian target of rapamycin
NAD: Nicotinamide adenine dinucleotide
NADH: Nicotinamide adenine dinucleotide hydride
OCR: Oxygen consumption rate
OXPHOS: Oxidative phosphorylation
PBS: Phosphate buffered saline
PC: Prostate cancer
PCR: Polymerase chain reaction
PI3K: Phosphoinositide 3-kinase
PSA: Prostate-specific antigen
PTEN: Phosphatase and tensin homolog
RHEB: Ras homolog enriched in brain
S6K1: Ribosomal protein S6 kinase 1
STAT: Signal transducer and activator of transcription
STAT3: Signal transducer and activator of transcription 3
t2DM: Type 2 diabetes mellitus
TBS: Tris-buffered saline
TCA: Tricarboxylic acid cycle
TIMP1: Tissue inhibitor of metalloproteinases 1
TMA: Tissue microarrays
TSC: Tuberous sclerosis complex
VIM: Vimentin
WT: Wild-type

## References

[1] Sung H, Ferlay J, Siegel RL, Laversanne M, Soerjomataram I, Jemal A, Bray F (2021). Global Cancer Statistics 2020: GLOBOCAN Estimates of Incidence and Mortality Worldwide for 36 Cancers in 185 Countries. CA Cancer J Clin.

[2] Carver BS, Chapinski C, Wongvipat J, Hieronymus H, Chen Y, Chandarlapaty S, Arora VK, Le C, Koutcher J, Scher H (2011). Reciprocal feedback regulation of PI3K and androgen receptor signaling in PTEN-deficient prostate cancer. Cancer Cell.

[3] Hirano T, Ishihara K, Hibi M (2000). Roles of STAT3 in mediating the cell growth, differentiation and survival signals relayed through the IL-6 family of cytokine receptors. Oncogene.

[4] Zhang HF (2014). STAT3 in Cancer-Friend or Foe?. Cancers.

[5] Priego N, Zhu L, Monteiro C, Mulders M, Wasilewski D, Bindeman W, Doglio L, Martinez L, Martinez-Saez E, Ramon YCS (2018). STAT3 labels a subpopulation of reactive astrocytes required for brain metastasis. Nat Med.

[6] Swoboda A, Soukup R, Eckel O, Kinslechner K, Wingelhofer B, Schorghofer D, Sternberg C, Pham HTT, Vallianou M, Horvath J (2021). STAT3 promotes melanoma metastasis by CEBP-induced repression of the MITF pathway. Oncogene.

[7] Ranger JJ, Levy DE, Shahalizadeh S, Hallett M, Muller WJ (2009). Identification of a Stat3-dependent transcription regulatory network involved in metastatic progression. Cancer Res.

[8] Grabner B, Schramek D, Mueller KM, Moll HP, Svinka J, Hoffmann T, Bauer E, Blaas L, Hruschka N, Zboray K (2015). Disruption of STAT3 signalling promotes KRAS-induced lung tumorigenesis. Nat Commun.

[9] Pencik J, Schlederer M, Gruber W, Unger C, Walker SM, Chalaris A, Marie IJ, Hassler MR, Javaheri T, Aksoy O (2015). STAT3 regulated ARF expression suppresses prostate cancer metastasis. Nat Commun.

[10] Oberhuber M, Pecoraro M, Rusz M, Oberhuber G, Wieselberg M, Haslinger P, Gurnhofer E, Schlederer M, Limberger T, Lagger S (2020). STAT3-dependent analysis reveals PDK4 as independent predictor of recurrence in prostate cancer. Mol Syst Biol.

[11] Demaria M, Giorgi C, Lebiedzinska M, Esposito G, D'Angeli L, Bartoli A, Gough DJ, Turkson J, Levy DE, Watson CJ (2010). A STAT3-mediated metabolic switch is involved in tumour transformation and STAT3 addiction. Aging (Albany NY).

[12] Wiebringhaus R, Pecoraro M, Neubauer HA, Trachtova K, Trimmel B, Wieselberg M, Pencik J, Egger G, Krall C, Moriggl R, et al: Proteomic Analysis Identifies NDUFS1 and ATP5O as Novel Markers for Survival Outcome in Prostate Cancer. Cancers. 2021;13(23):6036. 10.3390/cancers13236036. 10.3390/cancers13236036 PMC8656993 34885151

[13] Shaw RJ (2009). LKB1 and AMP-activated protein kinase control of mTOR signalling and growth. Acta Physiol (Oxf).

[14] Sonenberg N, Gingras AC (1998). The mRNA 5' cap-binding protein eIF4E and control of cell growth. Curr Opin Cell Biol.

[15] Zoncu R, Efeyan A, Sabatini DM (2011). mTOR: from growth signal integration to cancer, diabetes and ageing. Nat Rev Mol Cell Biol.

[16] Tanaka K, Babic I, Nathanson D, Akhavan D, Guo D, Gini B, Dang J, Zhu S, Yang H, De Jesus J (2011). Oncogenic EGFR signaling activates an mTORC2-NF-kappaB pathway that promotes chemotherapy resistance. Cancer Discov.

[17] Statz CM, Patterson SE, Mockus SM (2017). mTOR Inhibitors in Castration-Resistant Prostate Cancer: A Systematic Review. Target Oncol.

[18] Formisano L, Napolitano F, Rosa R, D'Amato V, Servetto A, Marciano R, De Placido P, Bianco C, Bianco R (2020). Mechanisms of resistance to mTOR inhibitors. Crit Rev Oncol Hematol.

[19] Faes S, Demartines N, Dormond O (2017). Resistance to mTORC1 Inhibitors in Cancer Therapy: From Kinase Mutations to Intratumoral Heterogeneity of Kinase Activity. Oxid Med Cell Longev.

[20] Li J, Zhang Y, Zheng N, Li B, Yang J, Zhang C, Xia G, Zhang M (2020). CREB activity is required for mTORC1 signaling-induced primordial follicle activation in mice. Histochem Cell Biol.

[21] Howell JJ, Hellberg K, Turner M, Talbott G, Kolar MJ, Ross DS, Hoxhaj G, Saghatelian A, Shaw RJ, Manning BD (2017). Metformin Inhibits Hepatic mTORC1 Signaling via Dose-Dependent Mechanisms Involving AMPK and the TSC Complex. Cell Metab.

[22] Decensi A, Puntoni M, Goodwin P, Cazzaniga M, Gennari A, Bonanni B, Gandini S (2010). Metformin and cancer risk in diabetic patients: a systematic review and meta-analysis. Cancer Prev Res (Phila).

[23] Hirsch HA, Iliopoulos D, Tsichlis PN, Struhl K (2009). Metformin selectively targets cancer stem cells, and acts together with chemotherapy to block tumor growth and prolong remission. Cancer Res.

[24] Yu H, Zhong X, Gao P, Shi J, Wu Z, Guo Z, Wang Z, Song Y (2019). The Potential Effect of Metformin on Cancer: An Umbrella Review. Front Endocrinol (Lausanne).

[25] Rothermundt C, Hayoz S, Templeton AJ, Winterhalder R, Strebel RT, Bartschi D, Pollak M, Lui L, Endt K, Schiess R (2014). Metformin in chemotherapy-naive castration-resistant prostate cancer: a multicenter phase 2 trial (SAKK 08/09). Eur Urol.

[26] Wegrzyn J, Potla R, Chwae YJ, Sepuri NB, Zhang Q, Koeck T, Derecka M, Szczepanek K, Szelag M, Gornicka A (2009). Function of mitochondrial Stat3 in cellular respiration. Science.

[27] Shackelford DB, Shaw RJ (2009). The LKB1-AMPK pathway: metabolism and growth control in tumour suppression. Nat Rev Cancer.

[28] Mehenni H, Lin-Marq N, Buchet-Poyau K, Reymond A, Collart MA, Picard D, Antonarakis SE (2005). LKB1 interacts with and phosphorylates PTEN: a functional link between two proteins involved in cancer predisposing syndromes. Hum Mol Genet.

[29] Hermanova I, Zuniga-Garcia P, Caro-Maldonado A, Fernandez-Ruiz S, Salvador F, Martin-Martin N, Zabala-Letona A, Nunez-Olle M, Torrano V, Camacho L, et al: Genetic manipulation of LKB1 elicits lethal metastatic prostate cancer. J Exp Med. 2020;217(6):e20191787. 10.1084/jem.20191787. 10.1084/jem.20191787 PMC7971141 32219437

[30] Poffenberger MC, Metcalfe-Roach A, Aguilar E, Chen J, Hsu BE, Wong AH, Johnson RM, Flynn B, Samborska B, Ma EH (2018). LKB1 deficiency in T cells promotes the development of gastrointestinal polyposis. Science.

[31] Ulz P, Perakis S, Zhou Q, Moser T, Belic J, Lazzeri I, Wolfler A, Zebisch A, Gerger A, Pristauz G (2019). Inference of transcription factor binding from cell-free DNA enables tumor subtype prediction and early detection. Nat Commun.

[32] Barbieri I, Pensa S, Pannellini T, Quaglino E, Maritano D, Demaria M, Voster A, Turkson J, Cavallo F, Watson CJ (2010). Constitutively active Stat3 enhances neu-mediated migration and metastasis in mammary tumors via upregulation of Cten. Cancer Res.

[33] Lefebvre-Legendre L, Salin B, Schaeffer J, Brethes D, Dautant A, Ackerman SH, di Rago JP (2005). Failure to assemble the alpha 3 beta 3 subcomplex of the ATP synthase leads to accumulation of the alpha and beta subunits within inclusion bodies and the loss of mitochondrial cristae in Saccharomyces cerevisiae. J Biol Chem.

[34] Bourouh M, Marignani PA. The Tumor Suppressor Kinase LKB1: Metabolic Nexus. Front Cell Dev Biol. 2022;10:Article 881297. 10.3389/fcell.2022.881297. 10.3389/fcell.2022.881297 PMC9097215 35573694

[35] Linher-Melville K, Singh G (2014). The transcriptional responsiveness of LKB1 to STAT-mediated signaling is differentially modulated by prolactin in human breast cancer cells. BMC Cancer.

[36] Shaw RJ, Bardeesy N, Manning BD, Lopez L, Kosmatka M, DePinho RA, Cantley LC (2004). The LKB1 tumor suppressor negatively regulates mTOR signaling. Cancer Cell.

[37] Taylor BS, Schultz N, Hieronymus H, Gopalan A, Xiao Y, Carver BS, Arora VK, Kaushik P, Cerami E, Reva B (2010). Integrative genomic profiling of human prostate cancer. Cancer Cell.

[38] Ollila S, Domenech-Moreno E, Laajanen K, Wong IP, Tripathi S, Pentinmikko N, Gao Y, Yan Y, Niemela EH, Wang TC (2018). Stromal Lkb1 deficiency leads to gastrointestinal tumorigenesis involving the IL-11-JAK/STAT3 pathway. J Clin Invest.

[39] Su Y, Zhang W, Patro CPK, Zhao J, Mu T, Ma Z, Xu J, Ban K, Yi C, Zhou Y (2020). STAT3 Regulates Mouse Neural Progenitor Proliferation and Differentiation by Promoting Mitochondrial Metabolism. Front Cell Dev Biol.

[40] Napoli E, Ross-Inta C, Wong S, Hung C, Fujisawa Y, Sakaguchi D, Angelastro J, Omanska-Klusek A, Schoenfeld R, Giulivi C (2012). Mitochondrial dysfunction in Pten haplo-insufficient mice with social deficits and repetitive behavior: interplay between Pten and p53. PLoS One.

[41] Dien J, Amin HM, Chiu N, Wong W, Frantz C, Chiu B, Mackey JR, Lai R (2006). Signal transducers and activators of transcription-3 up-regulates tissue inhibitor of metalloproteinase-1 expression and decreases invasiveness of breast cancer. Am J Pathol.

[42] Wu Y, Diab I, Zhang X, Izmailova ES, Zehner ZE (2004). Stat3 enhances vimentin gene expression by binding to the antisilencer element and interacting with the repressor protein, ZBP-89. Oncogene.

[43] Yao J, Qian CJ, Ye B, Zhao ZQ, Wei J, Liang Y, Zhang X (2014). Signal transducer and activator of transcription 3 signaling upregulates fascin via nuclear factor-kappaB in gastric cancer: Implications in cell invasion and migration. Oncol Lett.

[44] Giraud S, Bienvenu F, Avril S, Gascan H, Heery DM, Coqueret O (2002). Functional interaction of STAT3 transcription factor with the coactivator NcoA/SRC1a. J Biol Chem.

[45] Clements A, Gao B, Yeap SHO, Wong MKY, Ali SS, Gurney H (2011). Metformin in prostate cancer: two for the price of one. Ann Oncol.

[46] Pircher A, Zieher M, Eigentler A, Pichler R, Schafer G, Fritz J, Puhr M, Steiner E, Horninger W, Klocker H, Heidegger I (2018). Antidiabetic drugs influence molecular mechanisms in prostate cancer. Cancer Biol Ther.

[47] Mahoney SJ, Narayan S, Molz L, Berstler LA, Kang SA, Vlasuk GP, Saiah E (2018). A small molecule inhibitor of Rheb selectively targets mTORC1 signaling. Nat Commun.

[48] Ahn HK, Lee YH, Koo KC: Current Status and Application of Metformin for Prostate Cancer: A Comprehensive Review. Int J Mol Sci. 2020;21(22):8540. 10.3390/ijms21228540. 10.3390/ijms21228540 PMC7698147 33198356

[49] Wang Y, An H, Liu T, Qin C, Sesaki H, Guo S, Radovick S, Hussain M, Maheshwari A, Wondisford FE (2019). Metformin Improves Mitochondrial Respiratory Activity through Activation of AMPK. Cell Rep.

[50] He L, Wondisford FE (2015). Metformin action: concentrations matter. Cell Metab.

[51] Seim I, Jeffery PL, Thomas PB, Nelson CC, Chopin LK (2017). Whole-Genome Sequence of the Metastatic PC3 and LNCaP Human Prostate Cancer Cell Lines. G3 (Bethesda).

[52] Vial G, Detaille D, Guigas B (2019). Role of Mitochondria in the Mechanism(s) of Action of Metformin. Front Endocrinol (Lausanne).

[53] Madiraju AK, Erion DM, Rahimi Y, Zhang XM, Braddock DT, Albright RA, Prigaro BJ, Wood JL, Bhanot S, MacDonald MJ (2014). Metformin suppresses gluconeogenesis by inhibiting mitochondrial glycerophosphate dehydrogenase. Nature.

[54] Lahiri T, Brambilla L, Andrade J, Askenazi M, Ueberheide B, Levy DE (2021). Mitochondrial STAT3 regulates antioxidant gene expression through complex I-derived NAD in triple negative breast cancer. Mol Oncol.

[55] Arredouani MS, Lu B, Bhasin M, Eljanne M, Yue W, Mosquera JM, Bubley GJ, Li V, Rubin MA, Libermann TA, Sanda MG (2009). Identification of the transcription factor single-minded homologue 2 as a potential biomarker and immunotherapy target in prostate cancer. Clin Cancer Res.

[56] Lapointe J, Li C, Higgins JP, van de Rijn M, Bair E, Montgomery K, Ferrari M, Egevad L, Rayford W, Bergerheim U (2004). Gene expression profiling identifies clinically relevant subtypes of prostate cancer. Proc Natl Acad Sci U S A.

[57] Cancer Genome Atlas Research Network (2015). The molecular taxonomy of primary prostate cancer. Cell.

[58] Jain S, Lyons CA, Walker SM, McQuaid S, Hynes SO, Mitchell DM, Pang B, Logan GE, McCavigan AM, O'Rourke D (2018). Validation of a Metastatic Assay using biopsies to improve risk stratification in patients with prostate cancer treated with radical radiation therapy. Ann Oncol.

[59] Rhodes DR, Yu J, Shanker K, Deshpande N, Varambally R, Ghosh D, Barrette T, Pandey A, Chinnaiyan AM (2004). ONCOMINE: a cancer microarray database and integrated data-mining platform. Neoplasia.

[60] Li Q, Deng Q, Chao HP, Liu X, Lu Y, Lin K, Liu B, Tang GW, Zhang D, Tracz A (2018). Linking prostate cancer cell AR heterogeneity to distinct castration and enzalutamide responses. Nat Commun.

[61] de la Iglesia N, Konopka G, Puram SV, Chan JA, Bachoo RM, You MJ, Levy DE, Depinho RA, Bonni A (2008). Identification of a PTEN-regulated STAT3 brain tumor suppressor pathway. Genes Dev.

[62] Ding Z, Wu CJ, Chu GC, Xiao Y, Ho D, Zhang J, Perry SR, Labrot ES, Wu X, Lis R (2011). SMAD4-dependent barrier constrains prostate cancer growth and metastatic progression. Nature.

[63] Chen Z, Trotman LC, Shaffer D, Lin HK, Dotan ZA, Niki M, Koutcher JA, Scher HI, Ludwig T, Gerald W (2005). Crucial role of p53-dependent cellular senescence in suppression of Pten-deficient tumorigenesis. Nature.

[64] Liu Y, Marks K, Cowley GS, Carretero J, Liu Q, Nieland TJ, Xu C, Cohoon TJ, Gao P, Zhang Y (2013). Metabolic and functional genomic studies identify deoxythymidylate kinase as a target in LKB1-mutant lung cancer. Cancer Discov.

[65] Shaywitz AJ, Greenberg ME (1999). CREB: a stimulus-induced transcription factor activated by a diverse array of extracellular signals. Annu Rev Biochem.

[66] He L, Sabet A, Djedjos S, Miller R, Sun X, Hussain MA, Radovick S, Wondisford FE (2009). Metformin and insulin suppress hepatic gluconeogenesis through phosphorylation of CREB binding protein. Cell.

[67] Wang Y, Liu G, Tong D, Parmar H, Hasenmayer D, Yuan W, Zhang D, Jiang J (2015). Metformin represses androgen-dependent and androgen-independent prostate cancers by targeting androgen receptor. Prostate.

[68] Ulz P, Belic J, Graf R, Auer M, Lafer I, Fischereder K, Webersinke G, Pummer K, Augustin H, Pichler M (2016). Whole-genome plasma sequencing reveals focal amplifications as a driving force in metastatic prostate cancer. Nat Commun.

[69] Detre S, Saclani Jotti G, Dowsett M (1995). A "quickscore" method for immunohistochemical semiquantitation: validation for oestrogen receptor in breast carcinomas. J Clin Pathol.

[70] Schlederer M, Mueller KM, Haybaeck J, Heider S, Huttary N, Rosner M, Hengstschlager M, Moriggl R, Dolznig H, Kenner L (2014). Reliable quantification of protein expression and cellular localization in histological sections. PLoS One.

[71] Schlessinger K, Levy DE (2005). Malignant transformation but not normal cell growth depends on signal transducer and activator of transcription 3. Cancer Res.

[72] Eberl M, Klingler S, Mangelberger D, Loipetzberger A, Damhofer H, Zoidl K, Schnidar H, Hache H, Bauer HC, Solca F (2012). Hedgehog-EGFR cooperation response genes determine the oncogenic phenotype of basal cell carcinoma and tumour-initiating pancreatic cancer cells. EMBO Mol Med.

[73] Klein K, Witalisz-Siepracka A, Gotthardt D, Agerer B, Locker F, Grausenburger R, Knab VM, Bergthaler A, Sexl V (2021). T Cell-Intrinsic CDK6 Is Dispensable for Anti-Viral and Anti-Tumor Responses In Vivo. Front Immunol.

[74] Hauser C, Schuettengruber B, Bartl S, Lagger G, Seiser C (2002). Activation of the mouse histone deacetylase 1 gene by cooperative histone phosphorylation and acetylation. Mol Cell Biol.

[75] Tripathi SK, Chen Z, Larjo A, Kanduri K, Nousiainen K, Aijo T, Ricano-Ponce I, Hrdlickova B, Tuomela S, Laajala E (2017). Genome-wide Analysis of STAT3-Mediated Transcription during Early Human Th17 Cell Differentiation. Cell Rep.

[76] Koressaar T, Remm M (2007). Enhancements and modifications of primer design program Primer3. Bioinformatics.

[77] Roulhac PL, Ward JM, Thompson JW, Soderblom EJ, Silva M, Moseley MA, Jarvis ED (2011). Microproteomics: quantitative proteomic profiling of small numbers of laser-captured cells. Cold Spring Harb Protoc.

[78] Wang H, Qian WJ, Mottaz HM, Clauss TR, Anderson DJ, Moore RJ, Camp DG, Khan AH, Sforza DM, Pallavicini M (2005). Development and evaluation of a micro- and nanoscale proteomic sample preparation method. J Proteome Res.

[79] Cox J, Mann M (2008). MaxQuant enables high peptide identification rates, individualized p.p.b.-range mass accuracies and proteome-wide protein quantification. Nat Biotechnol.

[80] Cox J, Michalski A, Mann M (2011). Software lock mass by two-dimensional minimization of peptide mass errors. J Am Soc Mass Spectrom.

[81] Cox J, Hein MY, Luber CA, Paron I, Nagaraj N, Mann M (2014). Accurate proteome-wide label-free quantification by delayed normalization and maximal peptide ratio extraction, termed MaxLFQ. Mol Cell Proteomics.

[82] Wickham H: ggplot2 : Elegant Graphics for Data Analysis. In *Use R!,*, 2nd edition. pp. 1 online resource (XVI, 260 pages 232 illustrations, 140 illustrations in color. Cham: Springer International Publishing : Imprint: Springer,; 2016:1 online resource (XVI, 260 pages 232 illustrations, 140 illustrations in color.

[83] Colaprico A, Silva TC, Olsen C, Garofano L, Cava C, Garolini D, Sabedot TS, Malta TM, Pagnotta SM, Castiglioni I (2016). TCGAbiolinks: an R/Bioconductor package for integrative analysis of TCGA data. Nucleic Acids Res.

[84] Walker SM, Knight LA, McCavigan AM, Logan GE, Berge V, Sherif A, Pandha H, Warren AY, Davidson C, Uprichard A (2017). Molecular subgroup of primary prostate cancer presenting with metastatic biology. Eur Urol.

