# Supplementary material for: STAT3/LKB1 controls metastatic prostate cancer by regulating mTORC1/CREB pathway
Source: Mol Cancer. 2023 Aug 12;22:133. doi: 10.1186/s12943-023-01825-8 (PMC10422794; doi:10.1186/s12943-023-01825-8)
Supplement: Supplementary file 1 — Additional file 1: Supplementary Fig. 1. (A) Circos plot of STAT3 and PTEN deletions: inner circle TCGA (n=492) and outer circle plasma-DNA samples from advanced PCa patients (n=43). (B) Gross anatomy of representative prostates isolated at 19 weeks of age from WT, Ptenpc−/−, Ptenpc−/−Stat3pc−/− andPtenpc−/−Stat3C/+ mice. Scale bars, 10 mm. (C) Transmission electron microscopy (TEM) pictures of mitochondria from WT, Ptenpc−/−, Ptenpc−/−Stat3pc−/− and Ptenpc−/−Stat3C/+ prostates, 19 weeks of age mice. Red arrowheads show defected mitochondrial shape. Stat3 activation in Ptenpc−/−Stat3C/+ cells reversed defected mitochondrial phenotype. Supplementary Fig. 2. (A) Western blot analysis of STAT3, LKB1, p-4E-BP1, 4E-BP1, p-S6 and S6 expression in WT and Stat3KO MEFs. β-actin servesas a loading control. (B) qRT–PCR analysis of STAT3 and STK11 transcript levels in WT and Stat3KO MEFs (n=3 each). (C) qRT–PCR analysis of Stk11, Mo25, Strada and Ampkα mRNA expression in prostates of 19-week-old WT, Ptenpc−/−, Ptenpc−/−Stat3pc−/− and Ptenpc−/−Stat3C/+ (n=3 each). Data were analyzed by Student’s t-test and are shown as mean ± s.d. Supplementary Fig. 3. (A) The heatmap of murine PCa proteomics with significant enrichment of genes involved in regulation of oxidative phosphorylation. 19-week-old WT, Ptenpc−/−, Ptenpc−/−Stat3pc−/−and Ptenpc−/−Stat3C/+ mice (n=3). (B, C) Heatmap of murine PCa proteomics with significant enrichment of genes involved in regulation of glutamine metabolism and glycolysis. 19-week-old WT, Ptenpc−/−, Ptenpc−/−Stat3pc−/− and Ptenpc−/−Stat3C/+ mice (n=3). (D) IHC analysis of p-ACC and FASN inprostates from 19-week-old WT, Ptenpc−/−, Ptenpc−/− Stat3pc−/− and Ptenpc−/−Stat3C/+ mice. Scale bars, 100 μm. Bar graphs indicate percentage of cellspositive for p-ACC and FASN in prostates of 19-week-old WT, Ptenpc−/−, Ptenpc−/−Stat3pc−/− and Ptenpc−/−Stat3C/+. Protein levels quantification was done with HistoQuest software (n≥3). (E) Western blot analysis of AR and ST [file 12943_2023_1825_MOESM1_ESM.pdf]

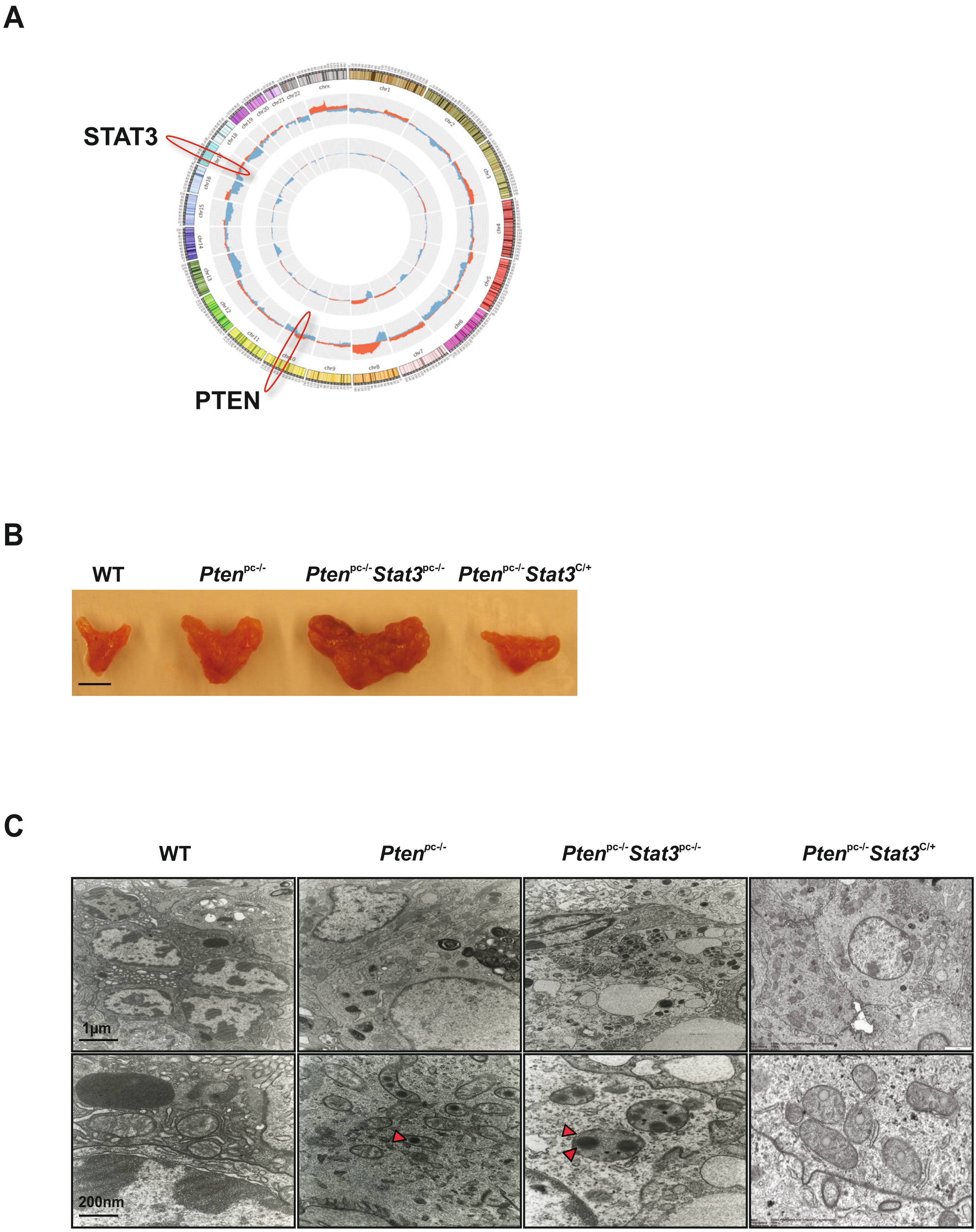

Pencik et al., Suppl. Figure 1

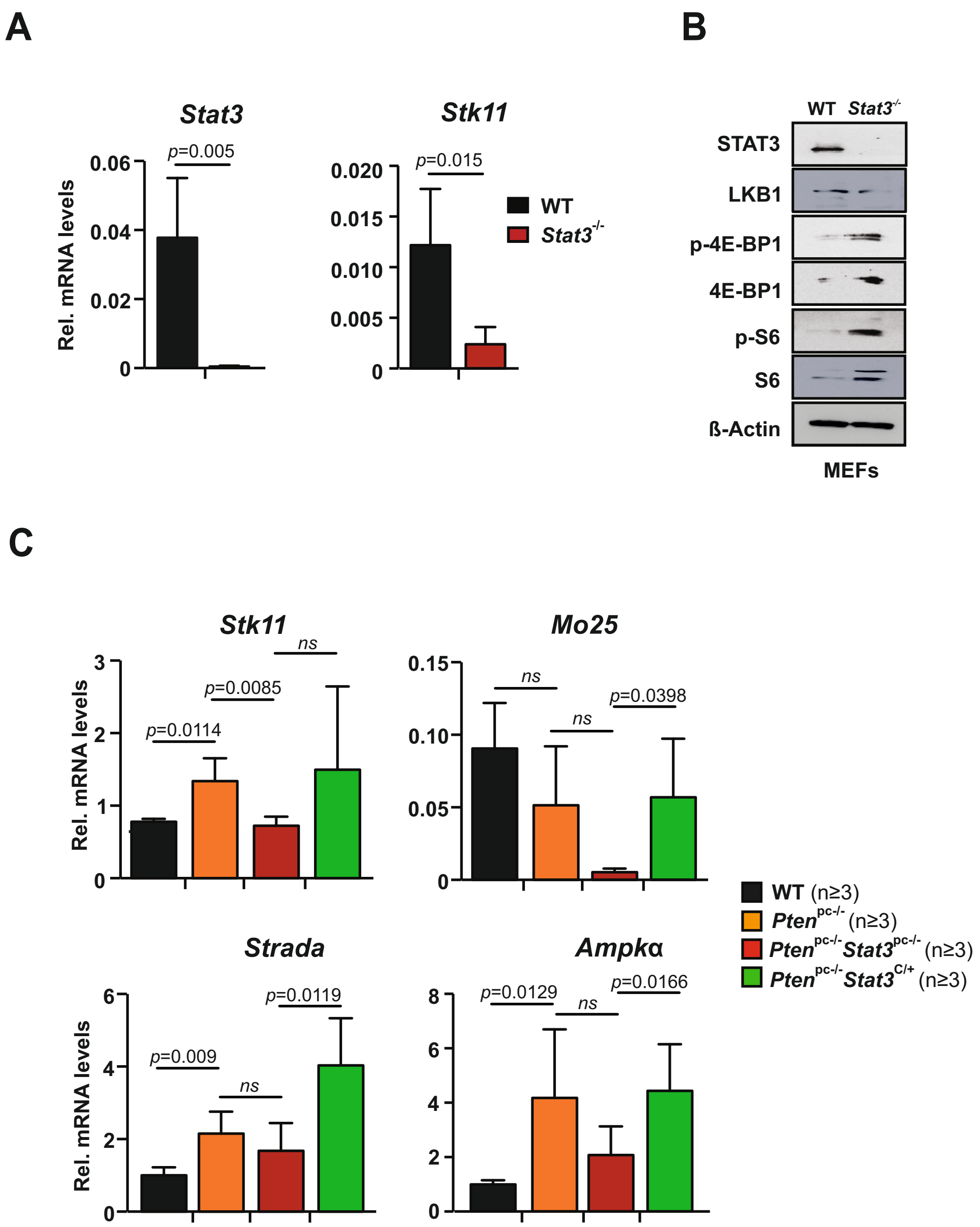

Pencik et al., Suppl. Figure 2

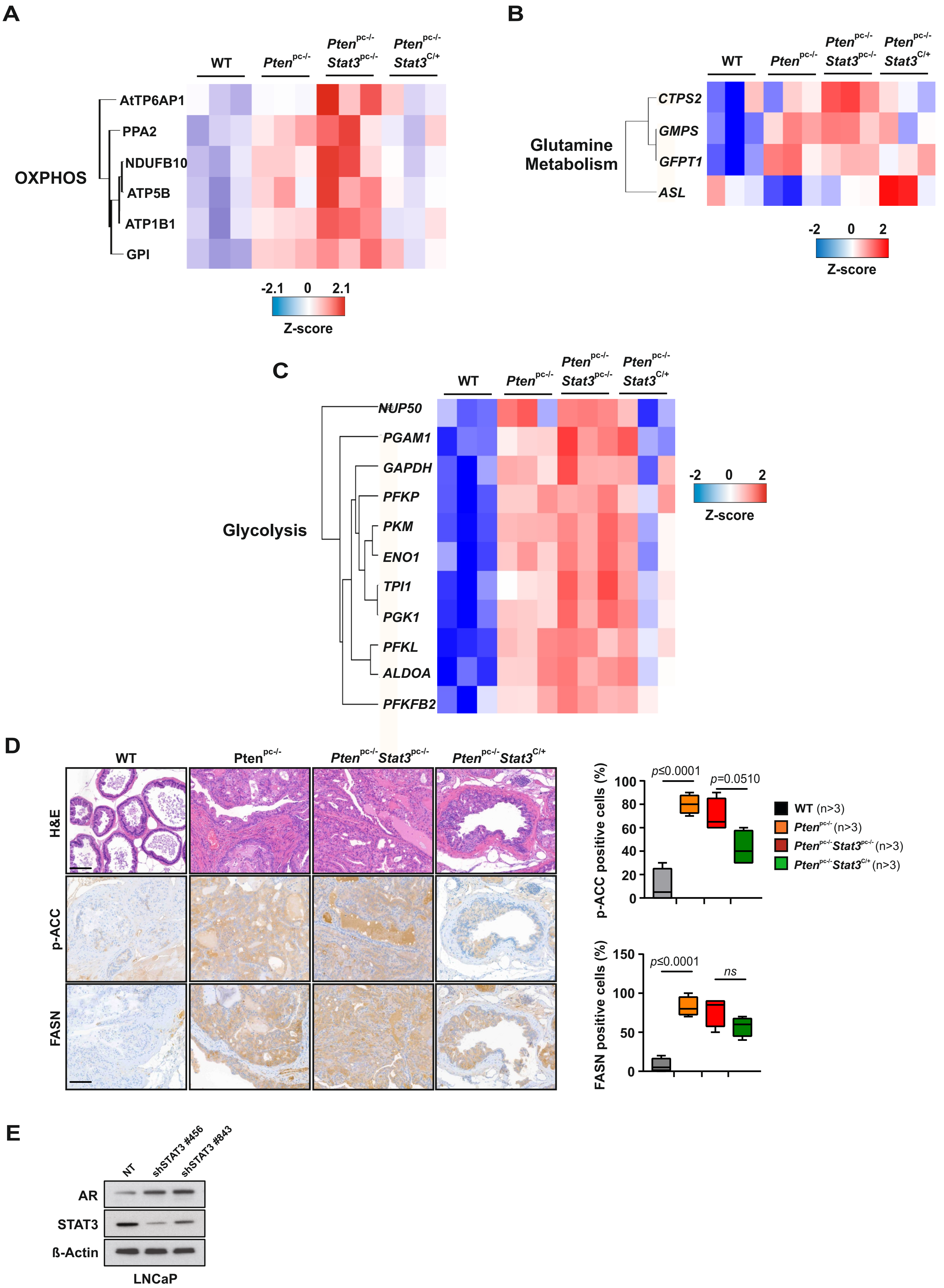

Pencik et al., Suppl. Figure 3

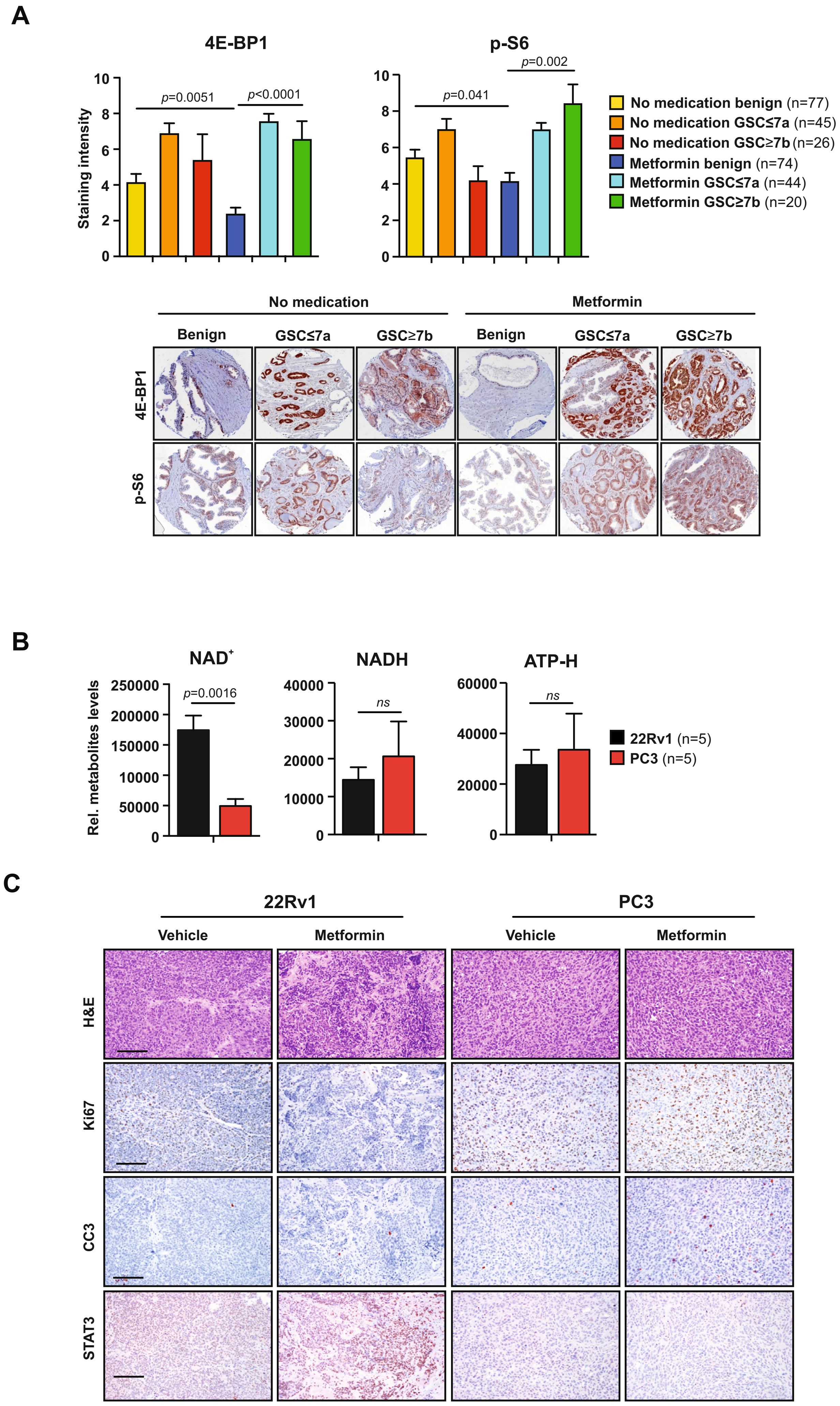

Pencik et al., Suppl. Figure 4

D

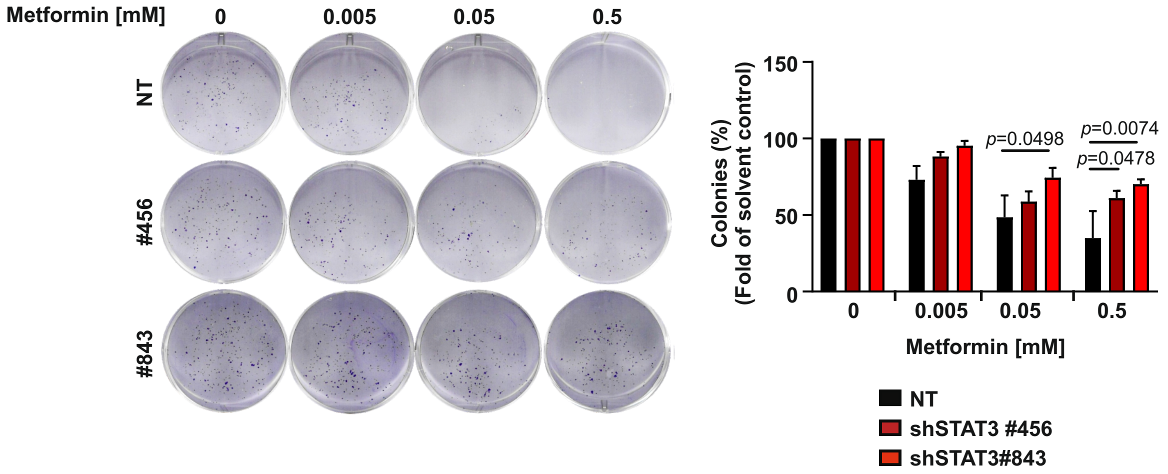

E

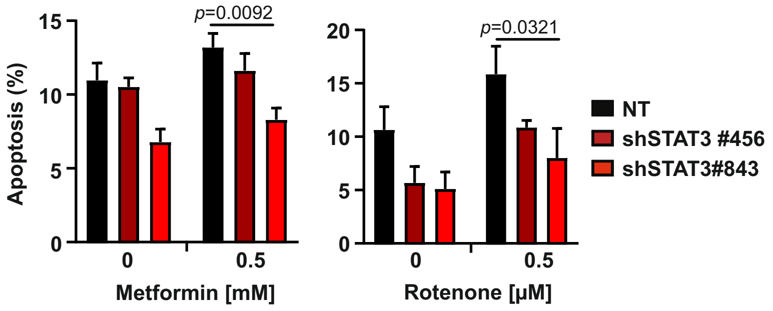

F

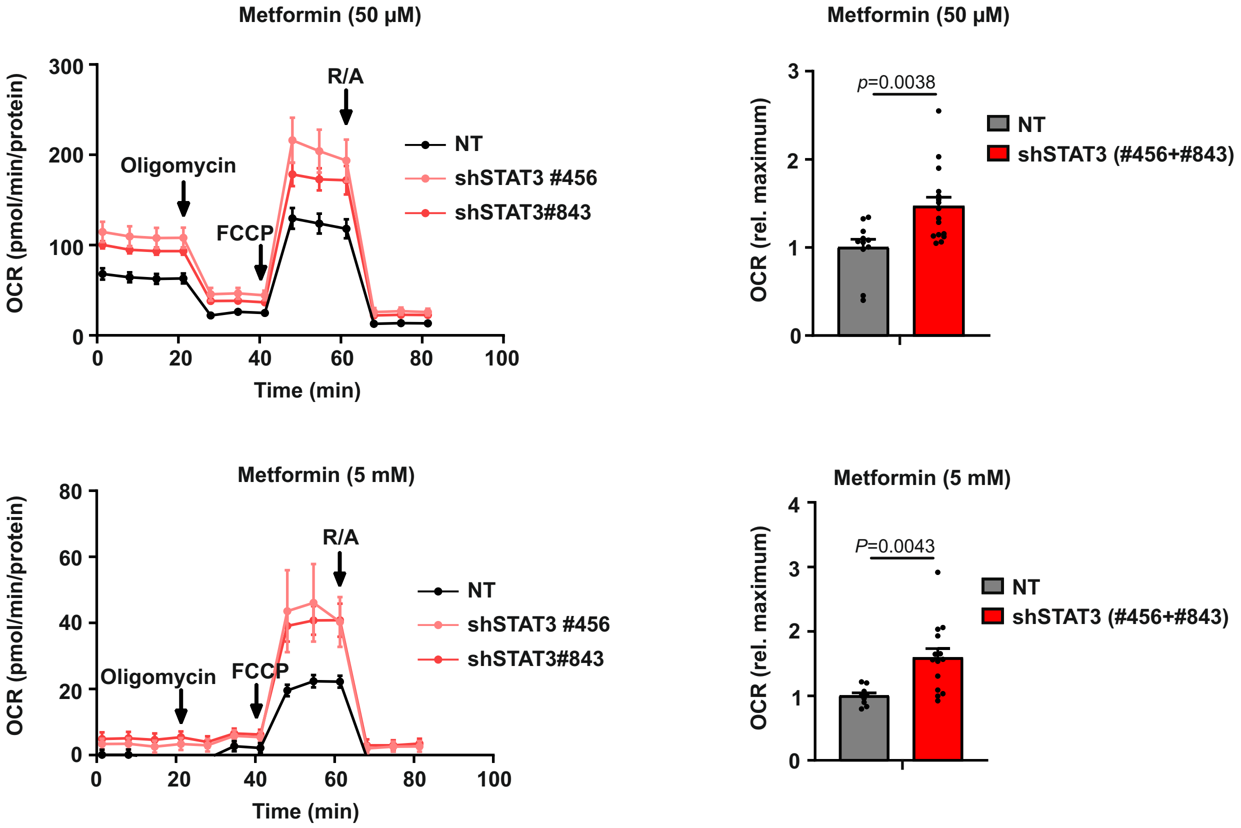

Pencik et al., Suppl. Figure 4

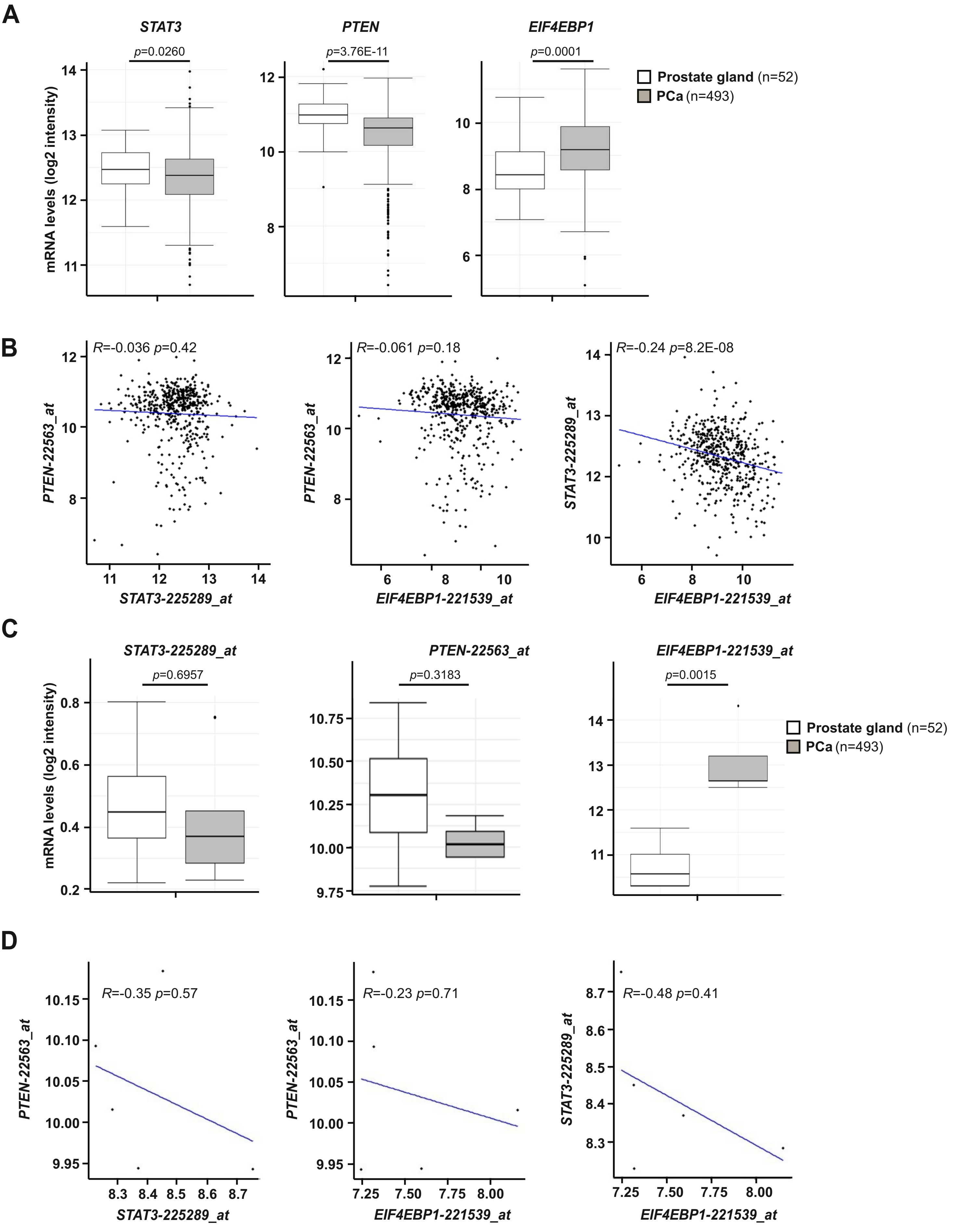

Pencik et al., Suppl. Figure 5

E

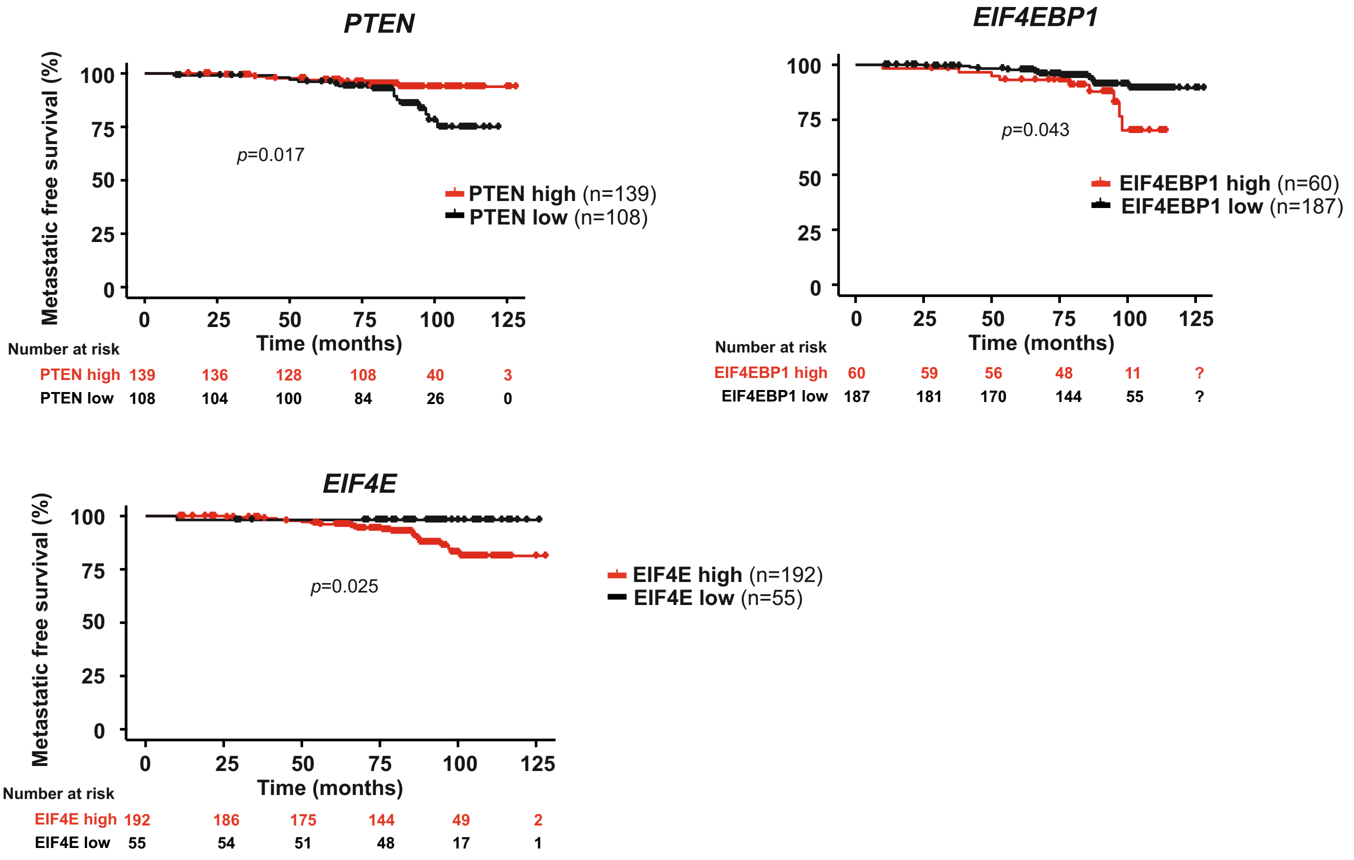

F

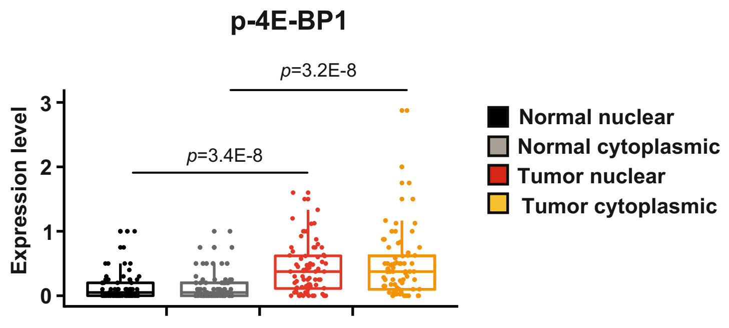

G

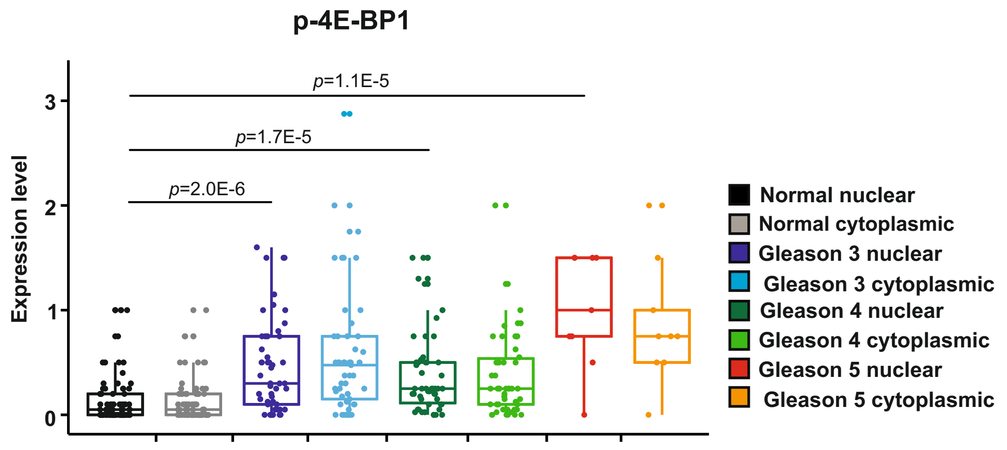

Pencik et al., Suppl. Figure 5

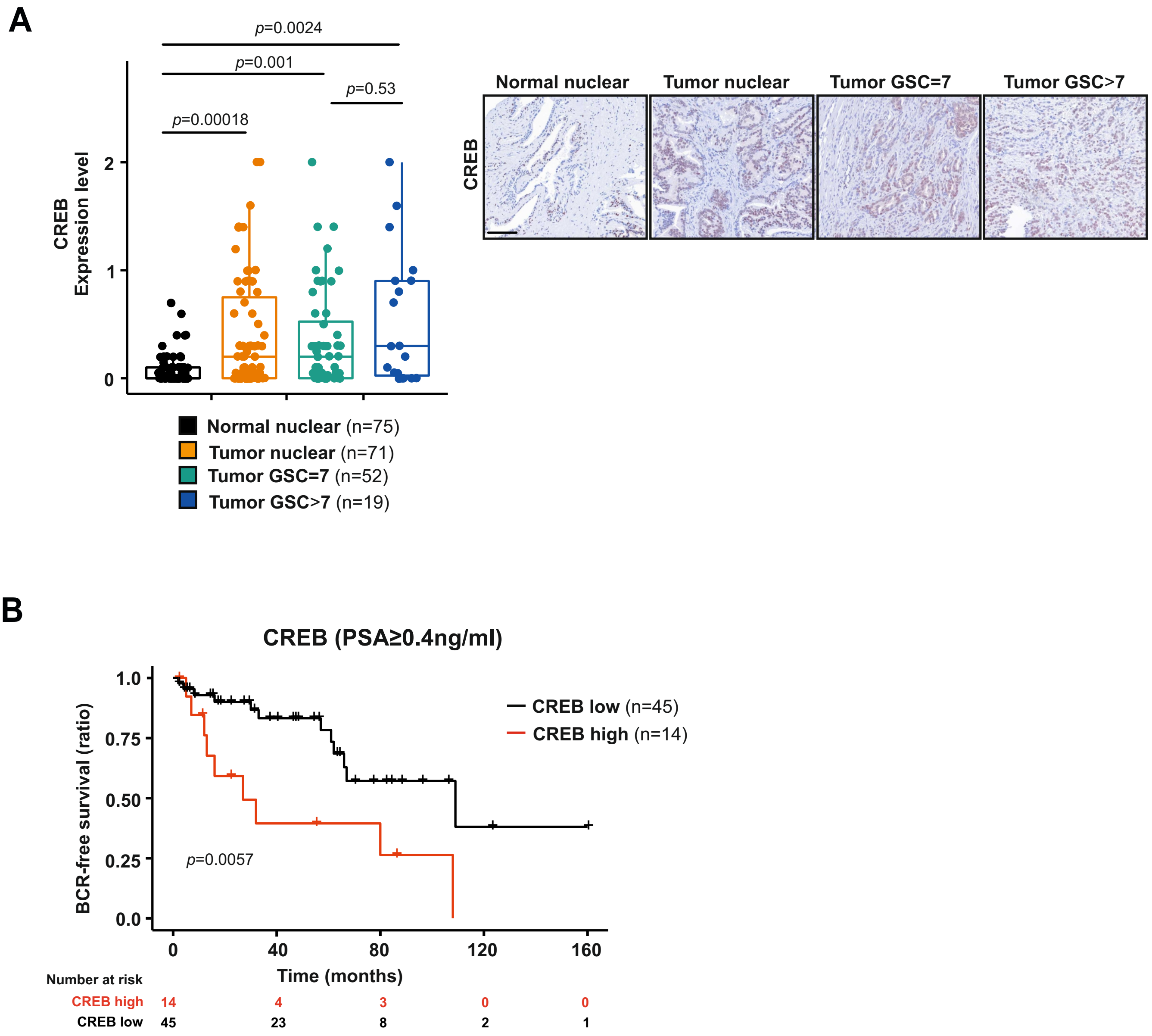

Pencik et al., Suppl. Figure 6

Supplementary Table 1

Human Primers qPCR		
Gene name	Forward 5‘-3‘	Reverse 5‘-3‘
STAT3	TGACATTCCCAAGGAGGAGGC	TGCAGCTTCCGTTCTCAGCTCC
**Mouse Primers qPCR**		
Gene name	Forward 5‘-3‘	Reverse 5‘-3‘
Stk11	CACACTTTACAACATCACCA	CTCATACTCCAACATCCCTC
Mo25	CCGTTCCCATTTGGCAAGTCT	GTCAAGCAGGTAGAGTGCCG
Strada	TTCCCTCTGTTCCCATGTCC	AGTCCCAGCCTTAACTGACC
AMPKα	CACCCTCACATCATCAAACTG	CTCCTCCAGAGACATATTCCA
**Primers ChIP qPCR**		
Gene name	Forward 5‘-3‘	Reverse 5‘-3‘
STK11_Pr1	GGACCTACCGATGCCAATTA	TGGGCAATAAGAGCGAAACT
STK11_Pr2	CTTTCCCCTGGTCCAAGAGT	GGCGAGGTCAAGGGTCAAAA
STK11_Pr1 Neg	GAGGACGAAGTTGACCCTGA	GAACACTTCCTCATGGACCTG
